# Identification of Functional Toxin/Immunity Genes Linked to Contact-Dependent Growth Inhibition (CDI) and Rearrangement Hotspot (Rhs) Systems

**DOI:** 10.1371/journal.pgen.1002217

**Published:** 2011-08-04

**Authors:** Stephen J. Poole, Elie J. Diner, Stephanie K. Aoki, Bruce A. Braaten, Claire t'Kint de Roodenbeke, David A. Low, Christopher S. Hayes

**Affiliations:** 1 Department of Molecular, Cellular, and Developmental Biology, University of California Santa Barbara, Santa Barbara, California, United States of America; 2 Biomolecular Science and Engineering Program, University of California Santa Barbara, Santa Barbara, California, United States of America; Environmental Research Institute, University College Cork, Ireland

## Abstract

Bacterial contact-dependent growth inhibition (CDI) is mediated by the CdiA/CdiB family of two-partner secretion proteins. Each CdiA protein exhibits a distinct growth inhibition activity, which resides in the polymorphic C-terminal region (CdiA-CT). CDI^+^ cells also express unique CdiI immunity proteins that specifically block the activity of cognate CdiA-CT, thereby protecting the cell from autoinhibition. Here we show that many CDI systems contain multiple *cdiA* gene fragments that encode CdiA-CT sequences. These “orphan” *cdiA-CT* genes are almost always associated with downstream *cdiI* genes to form *cdiA-CT/cdiI* modules. Comparative genome analyses suggest that *cdiA-CT/cdiI* modules are mobile and exchanged between the CDI systems of different bacteria. In many instances, orphan *cdiA-CT/cdiI* modules are fused to full-length *cdiA* genes in other bacterial species. Examination of *cdiA-CT/cdiI* modules from *Escherichia coli* EC93, *E. coli* EC869, and *Dickeya dadantii* 3937 confirmed that these genes encode functional toxin/immunity pairs. Moreover, the orphan module from EC93 was functional in cell-mediated CDI when fused to the N-terminal portion of the EC93 CdiA protein. Bioinformatic analyses revealed that the genetic organization of CDI systems shares features with *rhs* (rearrangement hotspot) loci. Rhs proteins also contain polymorphic C-terminal regions (Rhs-CTs), some of which share significant sequence identity with CdiA-CTs. All *rhs* genes are followed by small ORFs representing possible *rhsI* immunity genes, and several Rhs systems encode orphan *rhs-CT/rhsI* modules. Analysis of *rhs-CT/rhsI* modules from *D. dadantii* 3937 demonstrated that Rhs-CTs have growth inhibitory activity, which is specifically blocked by cognate RhsI immunity proteins. Together, these results suggest that Rhs plays a role in intercellular competition and that orphan gene modules expand the diversity of toxic activities deployed by both CDI and Rhs systems.

## Author Summary

Recent work from our laboratories has shown that many bacteria express contact-dependent growth inhibition (CDI) systems in which stick-like proteins on the cell surface deliver toxic tips into target cells. Over 60 distinct toxic tips have been identified in bacteria, and our data indicate that each CDI^+^ cell expresses a specific immunity protein that binds to its cognate toxin and inactivates it to prevent cell suicide. Here we identify genes for toxic tips that are not attached to the stick protein. Each of these “orphan” tips has toxic activity, which is blocked by its associated immunity protein. Remarkably, the orphan tips of some bacterial species are often found on the stick proteins in other species, suggesting that cells load and deliver different tips. We also report on a system called Rhs, which encodes another predicted stick-like protein that also carries variable tips. We found that the tips of Rhs proteins are toxic and that Rhs systems encode immunity proteins that specifically block toxin activity. CDI and Rhs toxin tip diversity may represent a microbial arms race, driven by the competition for environmental resources.

## Introduction

Many bacteria lead social lives in communities where they cooperate and compete with members of their own species, as well as those of other species [1]. One mechanism of bacterial communication is quorum sensing, in which small signaling molecules are released to coordinate group behavior when a critical cell density has been attained [2]. Other modes of communication based on direct cell-to-cell contact have recently been identified in bacteria. Contact-dependent signaling helps to coordinate cell aggregation and fruiting body formation in *Myxococcus xanthus* [3], and is also exploited to inhibit the growth of neighboring cells. Contact-dependent growth inhibition (CDI) was first described in the *Escherichia coli* isolate EC93 [4], and has subsequently been demonstrated in *Dickeya dadantii* 3937 [5]. CDI is mediated by the CdiB-CdiA two-partner secretion system. CdiB is a predicted outer membrane β-barrel protein that is required for secretion and presentation of the CdiA exoprotein on the cell surface [6], [7]. Like other two-partner secretion exoproteins, CdiA contains an N-terminal transport domain followed by a hemagglutinin repeat region that is predicted to adopt an extended filamentous β-helical structure [7]–[9]. The CDI growth inhibitory activity resides within the C-terminus of CdiA (CdiA-CT). The *cdi* locus also encodes a small CdiI immunity protein immediately downstream of *cdiA*. CdiI protects EC93 cells from CdiA-mediated growth autoinhibition [5].

CDI systems are widespread amongst α-, β-, and γ-proteobacteria [5]. CdiA exoproteins are related throughout most of their length, which varies from 1,400 to 2,000 amino acid residues in *Neisseria* and *Moraxella* species to over 5,600 residues for some *Dickeya* and *Pseudomonas* strains [5]. However, the CdiA-CT regions are highly variable, with CdiA sequences diverging abruptly after a VENN peptide motif found within the conserved DUF638 domain (Pfam PF04829). Similarly, CdiI sequences are also highly variable, suggesting that these immunity proteins specifically bind to cognate CdiA-CTs and neutralize their toxic activities. In support of this model, we recently showed that CdiA-CTs from *Dickeya dadantii* 3937 and uropathogenic *E. coli* (UPEC) 536 possess distinct toxic nuclease activities, and that the corresponding CdiI proteins bind to their cognate CdiA-CT and block nuclease activity both *in vitro* and *in vivo* [5]. Thus, CdiA-CT/CdiI pairs constitute a polymorphic family of toxin/immunity modules that allow CDI systems to deploy a wide variety of growth inhibition activities.

CdiA proteins share a number of characteristics with the Rhs protein family. The *rhs* genes were first identified in *E. coli* by C.W. Hill and colleagues, and were named rearrangement hotspots based on their role in chromosome duplications [10], [11]. Rhs proteins are widely distributed throughout the eubacteria, but their function is poorly understood. Like CdiA, Rhs proteins are large, ranging from ∼1,500 residues in Gram-negative bacteria to over 2,000 residues in some Gram-positive species. Rhs proteins also possess a central repeat region, though the characteristic YD peptide repeats of Rhs proteins are unrelated to the hemagglutinin repeats in CdiA. Moreover, Rhs proteins have variable C-terminal domains that are sharply demarcated by a conserved peptide motif (PxxxxDPxGL in the Enterobacteriaceae) [12]. Remarkably, we find that some CdiA and Rhs proteins share related C-terminal sequences, suggesting the protein families may be functionally analogous. Rhs proteins from a number of species appear to be exported to the cell surface [13]–[15], consistent with a role in cell-to-cell communication.

Here we show that many CDI systems have an unusual genetic organization similar to that described for some Rhs loci [12]. Downstream of the *cdiBAI* genes, CDI systems often contain fragmentary gene pairs that resemble *cdiA-CT/cdiI* toxin/immunity modules. The predicted *cdiA-CT* fragments generally lack translation initiation signals but encode the VENN peptide motif that demarcates the CdiA-CT region in full-length CdiA proteins. These “orphan” CdiA-CT proteins possess growth inhibitory activities, which are specifically neutralized by the corresponding orphan CdiI immunity proteins. Moreover, we show that the orphan *cdiA-CT/cdiI* region is actively transcribed in *E. coli* EC93. Although the orphan CdiA-CT does not appear to be synthesized, functional orphan CdiI immunity protein is produced in EC93. We also show that the Rhs systems of *D. dadantii* 3937 encode toxin/immunity pairs. Rhs-CTs from *D. dadantii* 3937 inhibit cell growth when expressed in *E. coli*, and this toxic activity is specifically neutralized by the cognate RhsI protein encoded immediately downstream. These results suggest that Rhs constitutes another class of cell-surface proteins involved in intercellular competition, and that orphan CT/immunity modules may represent a reservoir of toxin/immunity diversity for both CDI and Rhs systems.

## Results

### Many CDI Systems Contain Orphan *cdiA-CT/cdiI* Gene Pairs

**Figure 1:**
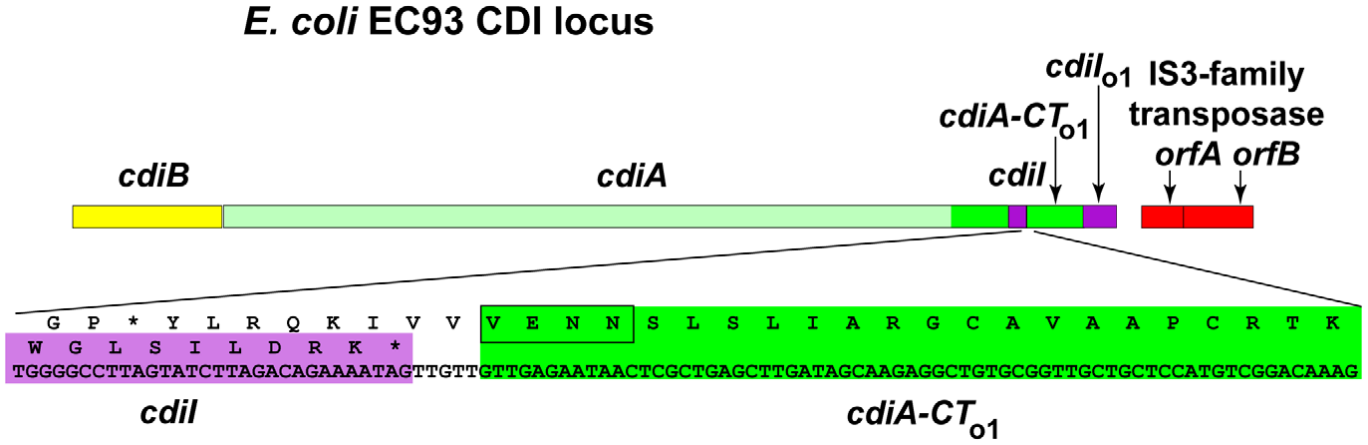
*E. coli* EC93 contains an orphan *cdiA-CT/cdiI* module. The CDI region of *E. coli* EC93 is depicted, with *cdiB*, *cdiA* and *cdiI* genes shown in yellow, green and purple, respectively. The *cdiA* coding region upstream of the encoded VENN motif is shown in light green, and the *cdiA-CT* sequence is shown in dark green. The orphan *cdiA-CT* fragment (*cdiA-CT*_o1_) and orphan *cdiI* (*cdiI*_o1_) are dark green and purple, respectively. The nucleotide sequence of the *cdiI - cdiA-CT*_o1_ junction and the predicted reading frames are shown in detail. Sequences similar to transposable element genes are shown in red.

Examination of the *cdi* locus in *E. coli* EC93 revealed two short open reading frames (ORFs) immediately downstream of the *cdiI* immunity gene (Figure 1). The first ORF lacks a translation initiation codon but encodes the VENN motif that typically demarcates variable CdiA-CT regions (Figure 1), suggesting the first ORF encodes a detached CdiA-CT remnant and the second ORF is its associated *cdiI* gene. A TBLASTN search of bacterial genomes revealed that the encoded proteins are related to the CdiA-CT/CdiI toxin/immunity pair from *E. coli* UPEC 536 (Figure S1). Thus, the *cdiBAI* gene cluster in *E. coli* EC93 is followed immediately by an “orphan” *cdiA-CT/cdiI* module related to the *cdi* locus of a different *E. coli* strain. To differentiate these modules from main *cdiBAI* clusters, we indicate orphan genes with a subscripted “o” and a number that indicates the position of the module in the *cdi* locus. Additionally, throughout the text we will indicate bacterial strains as superscripts. According to this nomenclature, the genes in the EC93 orphan module are designated *cdiA-CT*_o1_^EC93^ and *cdiI*_o1_^EC93^.

**Figure 2:**
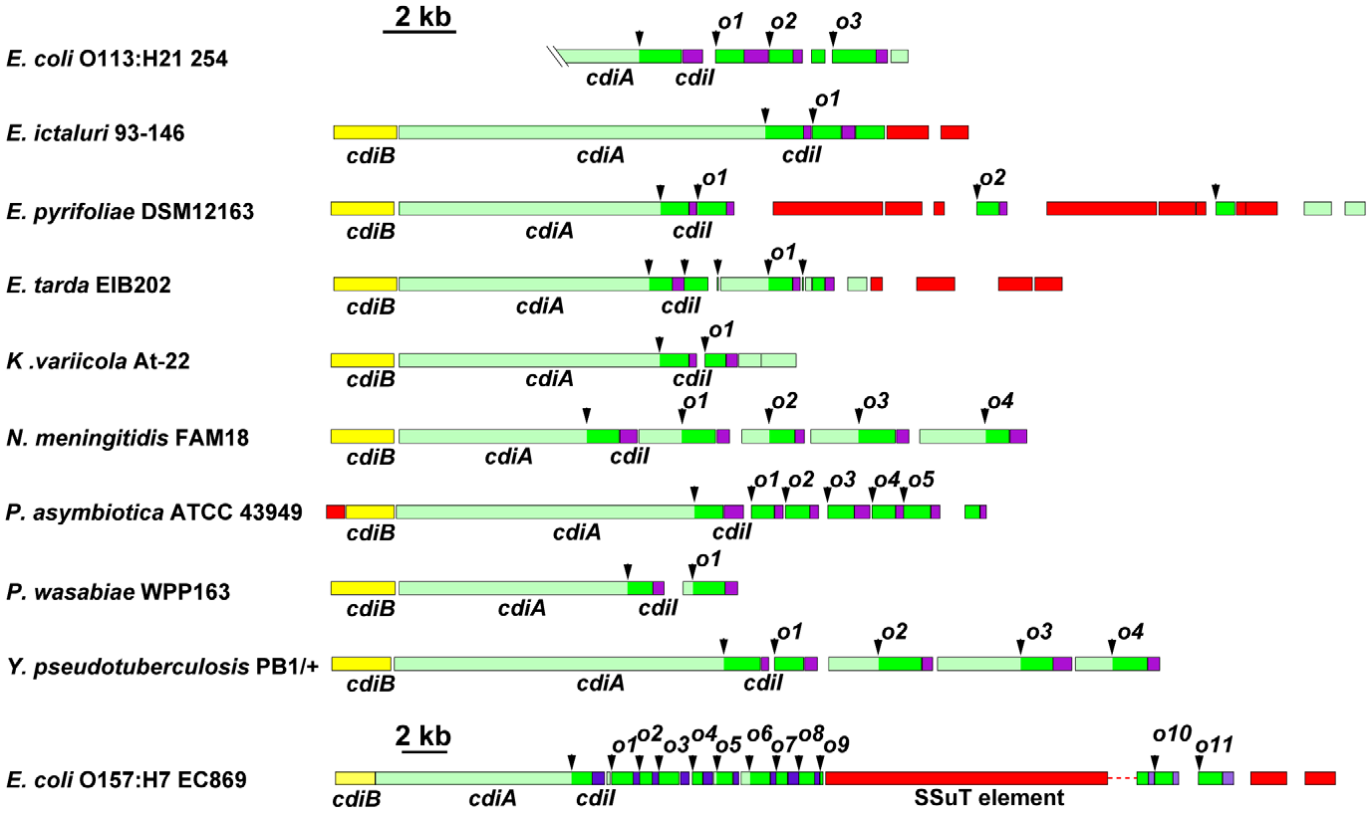
Many bacteria contain orphan *cdiA-CT/cdiI* modules. The *cdi* loci from selected bacterial species are presented using the color-coding scheme in Figure 1. Arrowheads indicate the positions of VENN encoding regions. Probable orphan *cdiA-CT/cdiI* modules are indicated with an “o” and numeral. Note the change in scale for the locus from *E. coli* EC869. The UniProt accession numbers for the encoded CdiA proteins are: *Escherichia coli* 254 ser. O113:H21 (A1XT91); *Edwardsiella ictaluri* 93–146 (C5BAK2); *Erwinia pyrifoliae* DSM 12163 (D2T668); *Edwardsiella tarda* EIB202 (D0ZDG0); *Klebsiella variicola* At-22 (D3RCW8); *Neisseria menigitidis* FAM18 (A1KSB8); *Photorhabdus asymbiotica* ATCC 43949 (B6VKN5); *Pectobacterium wasabiae* WPP163 (D0KFH4); *Yersinia pseudotuberculosis* PB1/+ (B2K3A8); and *Escherichia coli* EC869 (B3BM48). The *E. coli* 254 genomic sequence does not include the 5′-end of the *cdiA* gene.

Examination of CDI regions in other bacteria shows that orphan *cdiA-CT/cdiI* pairs are quite common. Although some CDI systems are comprised solely of the *cdiBAI* gene cluster, many loci are closely followed by one or more *cdiA* gene fragments that usually encode the VENN peptide motif (Figure 2). These orphan *cdiA-CT* genes are typically followed by small ORFs representing potential *cdiI* immunity genes. For example, the region II CDI system in *Yersinia pseudotuberculosis* PB1/+ contains four additional *cdiA-CT* gene fragments encoding the VENN motif, each associated with a putative *cdiI* gene (Figure 2 and Figure S2A). Three of these gene fragments (*cdiA-CT*_o2_^PB1(II)^, *cdiA-CT*_o3_^PB1(II)^ and *cdiA-CT*_o4_^PB1(II)^) share significant regions of homology with the upstream *cdiA* ^PB1(II)^ gene. The extent of these homologous regions varies between orphans, but the homology to full-length *cdiA* ^PB1(II)^ is limited to regions upstream of the VENN encoding sequences for *cdiA-CT*_o2_^PB1(II)^ and *cdiA-CT*_o3_^PB1(II)^ (Figure S2 and Figure S3). In contrast, the orphan *cdiA-CT*_o1_^PB1(II)^ gene shares no significant identity with the full-length *cdiA* ^PB1(II)^ gene beyond the sequence encoding the VENN peptide (Figure S2C). Downstream of the VENN encoding region, the orphan *cdiA-CT*_o_^PB1(II)^ genes are unrelated to one another, but have homology to *cdiA* genes and *cdiA-CT* fragments from other bacteria. The predicted orphan CdiA-CT_o1_^PB1(II)^ (UniProt B2K3A6) is related to CdiA-CTs from *E. coli* A0 34/86 (Q1RPM1; 87% identity over 107 residues) and *Enterobacter cloacae* subsp. *cloacae* ATCC 13047 (D5CBA0; 50% identity over 183 residues), as well as to orphan CdiA-CTs from *Dickeya zeae* Ech1591 (C6CGV6; 88% identity over 111 residues) and *Citrobacter rodentium* ICC168 (D2TJP2; 86% identity over 107 residues). Orphan CdiA-CT_o2_^PB1(II)^ (B2K3A4) is related to the CdiA-CT from *Serratia proteamaculans* 568 (A8GK56; 63% identity over 131 residues). Orphan CdiA-CT_o3_^PB1(II)^ (B2K3A2) is related to a CdiA-CT from *Erwinia amylovora* CFBP1430 (D4HWF3; 63% identity over 127 residues) and to orphan CdiA-CTs from *E. coli* EC869 (B3BM80; 75% identity over 297 residues) and *Neisseria meningitidis* MC58 (Q9K0T4; 57% identity over 136 residues). Finally, orphan CdiA-CT_o4_^PB1(II)^ (B2K3A0) is related to the CdiA-CT encoded by the adjacent *cdiA* ^PB1(II)^ gene (39% identity over 179 residues), and also to CdiA-CTs from *Klebsiella pneumoniae* 342 (B5Y0C2; 61% identity over 263 residues) and *Dickeya dadantii* Ech586 (D2BZ75; 59% identity over 263 residues).

Although some CDI systems, such as those in *Y. pseudotuberculosis* PB1/+ and *Neisseria meningitidis* FAM18, have well-ordered arrays of orphan *cdiA-CT* gene fragments downstream of *cdiBAI*, other species have more complex genomic arrangements. *Klebsiella variicola* At-22 and *Erwinia pyrifoliae* DSM 12163 both contain *cdiA-CT* fragments that do not encode VENN and lack associated *cdiI* genes (Figure 2). In several instances, the orphan *cdiA-CT* genes are disrupted by IS elements and transposon genes. For example, the orphan *cdiA-CT*_o9_^EC869^ from *E. coli* EC869 is interrupted by an SSuT antimicrobial resistance element [16]. Orphan *cdiA-CT* fragments can retain varying amounts of *cdiA* sequence upstream of the VENN-encoding region, but in some cases these homologous sequences are absent (Figure 2). For example, the orphan *cdiA-CT*_o7_^EC869^ gene from *E. coli* EC869 is unrelated to the adjacent *cdiA-CT*_o_^EC869^ fragments, but is almost identical (99% identity from the VENN region onward) to the orphan *cdiA-CT*_o1_^254^ from *E. coli* strain 254 (Figure S4). Moreover, the associated *cdiI* immunity genes are also nearly identical. A comparison of the *E. coli* EC869 and *E. coli* strain 254 genomes shows that the homology between these orphan *cdiA-CT/cdiI* modules begins at the VENN encoding sequence and extends precisely to the VENN sequence of the next *cdiA-CT* orphan (Figure S4).

### Interchange of *cdiA-CT/cdiI* Modules

**Figure 3:**
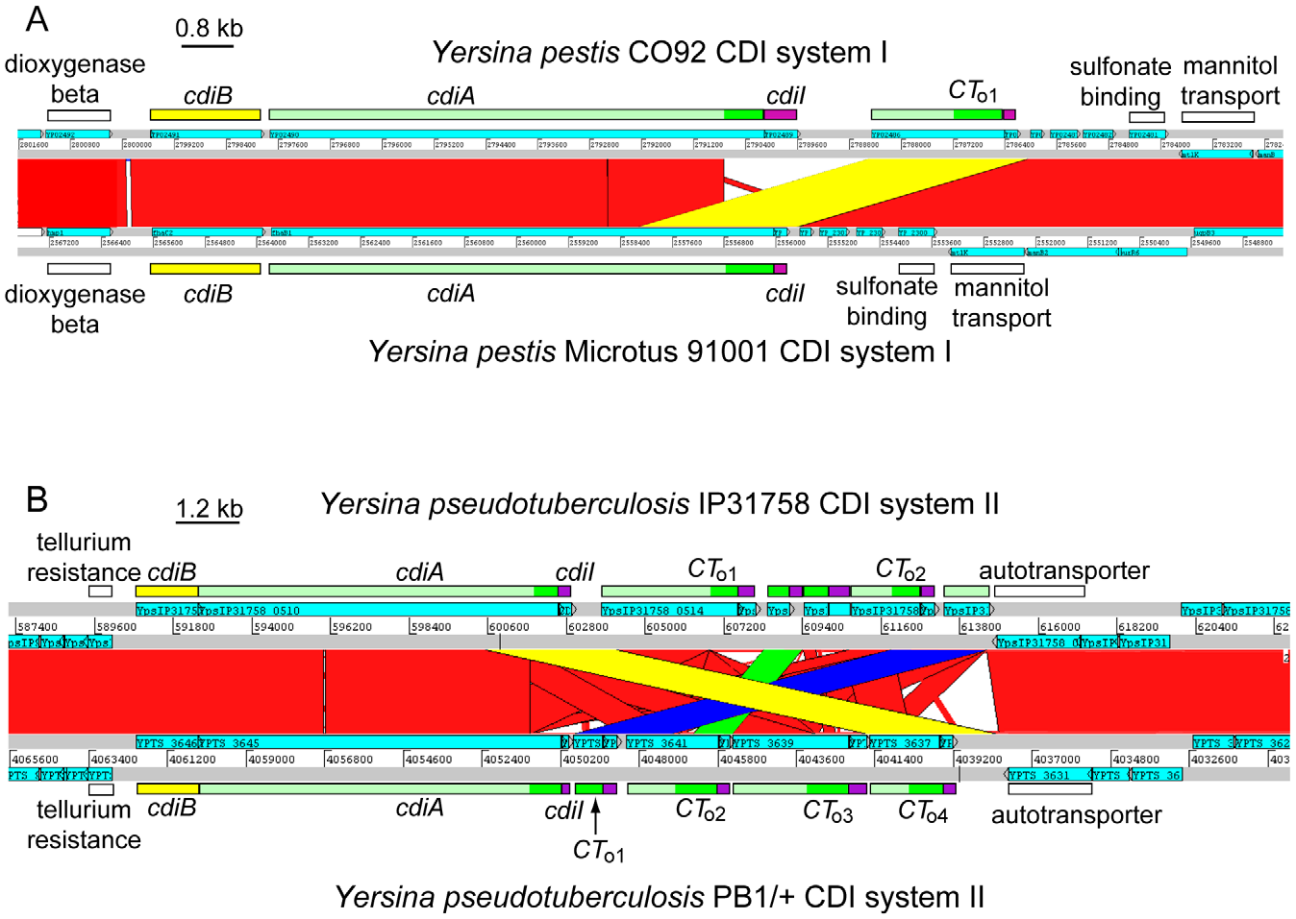
Interchange between *cdiA* and orphan *cdiA-CT*s. A) ACT comparison of the CDI systems (region I) from *Y. pestis* CO92 (top) and *Y. pestis* Microtus 91001 (bottom). The *cdi* loci lie within a highly conserved genomic region (red blocks denote nucleotide conservation), but the sequences encoding CdiA-CT diverge beginning at the VENN region. A 3,557 bp deletion in *Y. pestis* Microtus 91001 has fused the orphan *cdiA-CT/cdiI* module to the full-length *cdiA* gene (highlighted in yellow). B) ACT comparison of region II *cdi* loci from *Y. pseudotuberculosis* strains IP31758 (top) and PB1/+ (bottom). The *cdi* loci lie within a highly conserved genomic region (red denotes nucleotide conservation), but the *cdiA-CT* sequences exhibit complex rearrangements between the two strains. The orphan *cdiA-CT*_o4_^PB1(II)^/*cdiI*_o4_^PB1(II)^ module of strain PB1/+ is essentially identical to the *cdiA-CT* ^IP31758(II)^ and *cdiI* ^IP31758(II)^ sequences from strain IP31758 (99.1% identity over 3,648 nucleotides; highlighted in yellow). Additionally, the orphan *cdiA-CT*_o1_^PB1(II)^/*cdiI*_o1_^PB1(II)^ module is nearly identical to the orphan *cdiA-CT*_o2_^IP31758(II)^/*cdiI*_o2_^IP31758(II)^ module (98% identity over 2,656 nucleotides, highlighted in blue), and the *cdiA-CT*_o2_^PB1(II)^/*cdiI*_o2_^PB1(II)^ module is nearly identical to a *cdiA* fragment (and associated *cdiI* gene) found between the two orphan modules in strain IP31758 (98% identity over 1,073 nucleotides; highlighted in green).

All sequenced *Y. pestis* strains share two large blocks of conserved DNA that contain *cdi* loci. For the region I CDI system (positioned between the mannitol transporter and dioxygenase β-subunit genes), the predicted CdiA^(I)^ protein is identical in all fully sequenced *Y. pestis* strains with the exception of the Microtus 91001 strain. CdiA^(I)^ sequences N-terminal to the VENN motif are essentially identical in Microtus 91001 and other *Y. pestis* strains, though there is a six amino acid residue deletion within the hemagglutinin repeat region of Microtus 91001. However, following the VENN motif, the CdiA-CT^91001(I)^ of Microtus 91001 diverges from that of other *Y. pestis* strains (Figure S5). Pairwise comparison of the *Y. pestis* CO92 and *Y. pestis* Microtus 91001 genomes revealed a 3,557 base-pair deletion in the Microtus 91001 region I *cdi* locus that has fused an orphan *cdiA-CT/cdiI* module to the upstream *cdiA* ^91001(I)^ gene (Figure 3A), producing a CdiA/CdiI toxin/immunity pair that is different from other *Y. pestis* strains. Thus, the CdiA^91001(I)^ protein from Microtus 91001 contains an orphan CdiA-CT effector domain from the CO92 strain.

Another possible example of CdiA-CT interchange is found in the region II CDI system (located between tellurium resistance genes and a predicted autotransporter) shared by *Y. pseudotuberculosis* PB1/+ and IP31758 strains. The main *cdiA-CT* ^IP31758(II)^ and *cdiI* ^IP31758(II)^ sequences of *Y. pseudotuberculosis* IP31758 are essentially identical to the *cdiA-CT*_o4_^PB1(II)^/*cdiI*_o4_^PB1(II)^ orphan module from strain PB1/+ (99% identity over 3,646 nucleotides) (Figure 3B). Additionally, comparison of these loci revealed other complex rearrangements. The orphan *cdiA-CT*_o1_^PB1(II)^/*cdiI*_o1_^PB1(II)^ module of strain PB1/+ is nearly identical to *cdiA-CT*_o2_^IP31758(II)^/*cdiI*_o2_^IP31758(II)^ from IP31758 (98% identity over 2,656 nucleotides), and the orphan *cdiA-CT*_o2_^PB1(II)^/*cdiI*_o2_^PB1(II)^ from PB1/+ is 98% identical (over 1,073 nucleotides) to a *cdiA* gene fragment (which lacks the VENN encoding sequence) and its associated *cdiI* gene in strain IP31758 (Figure 3B). There are at least two possible explanations for these observations. The two *Y. pseudotuberculosis* strains could have independently acquired the same modules and incorporated them at different sites within the *cdi* locus. Alternatively, the *cdi* locus of a common ancestor may have rearranged during strain diversification. Although the mechanisms underlying these complex exchanges are unknown, these observations suggest that orphan *cdiA-CT* fragments are a potential source of CdiA/CdiI toxin/immunity diversity.

### Orphan *cdiA-CT/cdiI* Modules Are Functional

**Figure 4:**
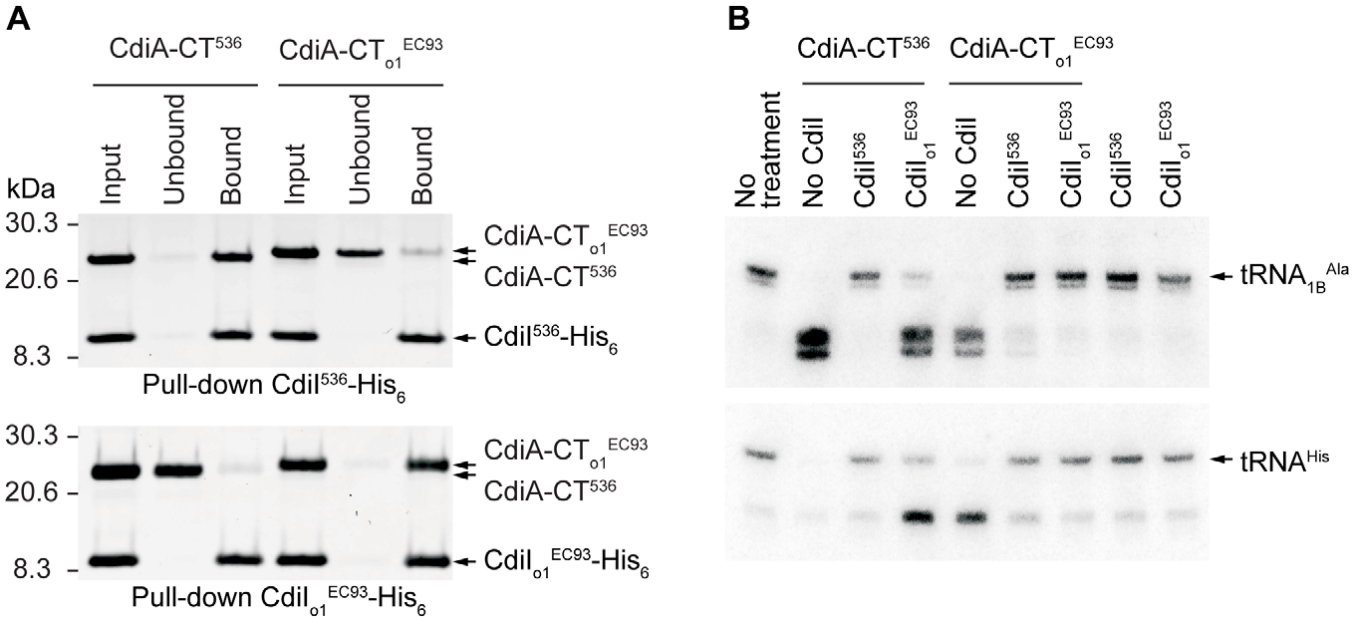
The tRNase activity of CdiA-CT_o1_^EC93^ is blocked by the binding of CdiI_o1_^EC93^. A) Analysis of CdiA-CT/CdiI binding. Purified CdiA-CT and CdiI-His_6_ proteins were mixed at equimolar ratios then purified by Ni^2+^-affinity chromatography. Input samples represent the protein mixtures prior to chromatography. Unbound fractions contain proteins that failed to bind the affinity resin. Bound proteins were eluted from the affinity resin with imidazole. All fractions were analyzed by SDS-PAGE. B) Northern blot analysis of CdiA-CT^UPEC536^ and CdiA-CT_o1_^EC93^ tRNase activity. S100 fractions containing cellular tRNA was treated with purified CdiA-CT and/or CdiI-His_6_ proteins and then analyzed by Northern blot hybridization using probes specific for tRNA_1B_^Ala^ and tRNA^His^.

If orphan *cdiA-CT/cdiI* modules are merely unused, nonfunctional remnants of full-length *cdiA/cdiI* genes, then there should be no selective pressure to maintain CdiA-CT toxin activity and CdiI immunity function. To determine whether orphan *cdiA-CT/cdiI* modules encode functional proteins, we characterized the orphan CdiA-CT/CdiI proteins from *E. coli* EC93. As described above, CdiA-CT_o1_^EC93^ and CdiI_o1_^EC93^ are very similar to the UPEC 536 CdiA-CT^UPEC536^/CdiI^UPEC536^ pair that we have previously characterized [5]. We first tested whether CdiA-CT_o1_^EC93^ and CdiI_o1_^EC93^ bind to one another as predicted for a toxin/immunity pair. We introduced a translation initiation signal upstream of the *cdiA-CT*_o1_^EC93^ fragment and co-expressed orphan CdiA-CT_o1_^EC93^ with CdiI_o1_^EC93^ immunity protein carrying a hexa-histidine (His_6_) epitope tag at its C-terminus. Ni^2+^-affinity purification of His_6_-tagged CdiI_o1_^EC93^ under non-denaturing conditions resulted in co-purification of CdiA-CT_o1_^EC93^ (data not shown), demonstrating that the two proteins bind each other. Given the similarity between CdiA-CT_o1_^EC93^ and CdiA-CT^UPEC536^, we next tested whether the CdiI^UPEC536^ and CdiI_o1_^EC93^ immunity proteins are able to bind near-cognate CdiA-CTs. His_6_-tagged CdiI proteins were first separated from their cognate CdiA-CT proteins by Ni^2+^-affinity chromatography under denaturing conditions. The individual proteins were then refolded and tested for binding to their cognate partners. Refolded CdiA-CT_o1_^EC93^ and CdiA-CT^UPEC536^ were able re-bind to their cognate CdiI proteins (Figure 4A). However, the His_6_-tagged CdiI proteins were unable to bind stably to the near-cognate CdiA-CTs (Figure 4A). These results show that the EC93 orphan CdiA-CT/CdiI proteins physically interact with one another, but appear to have diverged enough from the UPEC 536 system that high-affinity binding between the two systems no longer occurs.

We previously demonstrated that CdiA-CT^UPEC536^ is a nuclease that cleaves tRNA [5]; therefore we asked whether CdiA-CT_o1_^EC93^ also possesses this biochemical activity. Purified CdiA-CT_o1_^EC93^ cleaved a number of different tRNA species in a manner that was indistinguishable from CdiA-CT^UPEC536^ tRNase activity (Figure 4B and data not shown). This tRNase activity was inhibited by the addition of equimolar His_6_-tagged CdiI_o1_^EC93^ (Figure 4B). Intriguingly, His_6_-tagged CdiI^UPEC536^ was also able to neutralize the tRNase activity of CdiA-CT_o1_^EC93^, but CdiI_o1_^EC93^ had no effect on CdiA-CT^UPEC536^ activity (Figure 4B). Presumably, CdiI^UPEC536^ interacts with CdiA-CT_o1_^EC93^, but this binding is not of sufficient affinity to allow co-purification by Ni^2+^-affinity chromatography. Together, these results show that the EC93 orphan CdiA-CT/CdiI proteins retain the biochemical features of a functional toxin/immunity module.

### Orphan CdiA-CTs Inhibit Cell Growth

**Figure 5:**
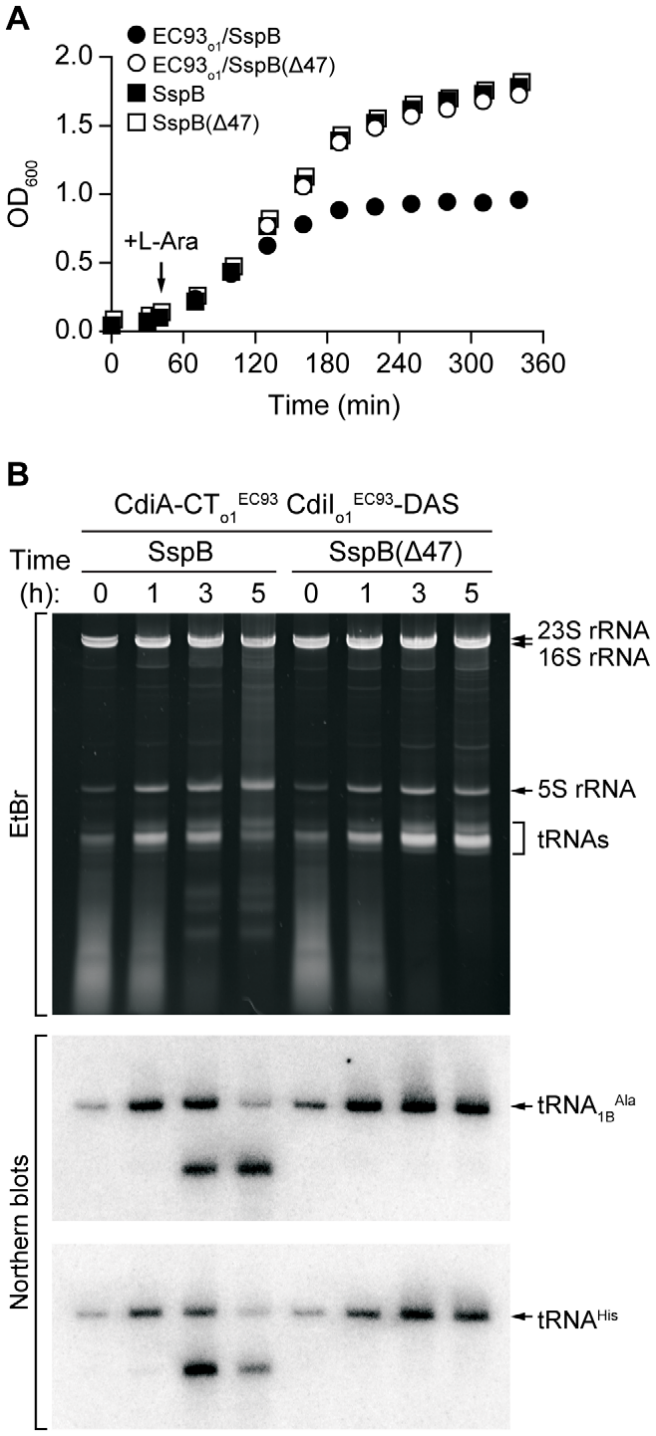
CdiA-CT_o1_^EC93^ inhibits the growth of *E. coli* cells. A) Growth curves of *E. coli* Δ*sspB* cells expressing CdiA-CT_o1_^EC93^/CdiI_o1_^EC93^-DAS. Degradation of CdiI_o1_^EC93^-DAS was initiated by the addition of L-arabinose to induce SspB synthesis. Control cells express SspB(Δ47), which does not deliver CdiI_o1_^EC93^-DAS to the ClpXP protease. Growth curves with square symbols represent control strains expressing SspB or SspB(Δ47), but not CdiA-CT_o1_^EC93^/CdiI_o1_^EC93^-DAS. B) Analysis of *in vivo* CdiA-CT_o1_^EC93^ tRNase activity. Total RNA was isolated from cells expressing CdiA-CT_o1_^EC93^/CdiI_o1_^EC93^-DAS at varying times after L-arabinose induction. Samples were run on polyacrylamide gels followed by staining with ethidium bromide (EtBr) or Northern blot analysis using probes specific for tRNA_1B_^Ala^ and tRNA^His^.

We next asked whether the EC93 orphan CdiA-CT retains growth inhibitory activity. In general, *cdiA-CT* gene fragments are very toxic and can only be maintained on plasmids if the cognate *cdiI* immunity gene is also present. However, CdiI immunity proteins efficiently block CdiA-CT activity, making it difficult to assess CdiA-CT toxicity. To circumvent this problem, we used the controllable proteolysis system of McGinness and Sauer [17] to activate CdiA-CT_o1_^EC93^ through degradation of the CdiI_o1_^EC93^ immunity protein. This strategy uses the SspB adaptor protein to deliver ssrA(DAS) peptide-tagged proteins to the ClpXP protease. We tagged the C-terminus of CdiI_o1_^EC93^ with the ssrA(DAS) peptide and co-expressed it with CdiA-CT_o1_^EC93^ in *E. coli* Δ*sspB* cells. Degradation of tagged CdiI_o1_^EC93^ was then initiated by induction of SspB synthesis from a plasmid-borne arabinose-inducible promoter, resulting in growth arrest after approximately 2 hours (Figure 5A). In contrast, growth continued unabated upon induction of SspB(Δ47) (Figure 5A), which lacks the C-terminal motif required for binding to ClpXP. Analysis of total cellular RNA revealed cleavage of tRNAs in the cells expressing wild-type SspB, but not in those expressing SspB(Δ47) (Figure 5B). These results demonstrate that CdiA-CT_o1_^EC93^ activity is unmasked upon CdiI_o1_^EC93^ degradation. Additionally, the temporal correlation between growth arrest and tRNA cleavage strongly suggests that the tRNase activity of CdiA-CT_o1_^EC93^ is responsible for growth inhibition.

To test whether other orphan *cdiA-CT/cdiI* modules have growth inhibition activity, we examined orphan gene pairs from *Dickeya dadantii* 3937 and *E. coli* EC869. *D. dadantii* 3937 contains one orphan *cdiA-CT/cdiI* module in the region I *cdi* locus. We cloned this module and added a C-terminal His_6_ epitope tag onto the predicted CdiI_o1_^3937^ protein. Overproduced CdiA-CT_o1_^3937^ protein co-purified with CdiI_o1_^3937^-His_6_ during Ni^2+^-affinity chromatography, indicating a binding interaction between these proteins (Figure S6A). Moreover, CdiA-CT_o1_^3937^ inhibited the growth of *E. coli* cells upon degradation of ssrA(DAS)-tagged CdiI_o1_^3937^ (Figure S6B). Examination of the orphan *cdiA-CT*_o11_ */cdiI*_o11_ module from *E. coli* EC869 gave similar results, except growth inhibition was delayed compared to the *D. dadantii* 3937 orphan system (Figure S6). These data demonstrate that other orphan *cdiA-CT/cdiI* modules also encode functional toxin/immunity pairs.

**Figure 6:**
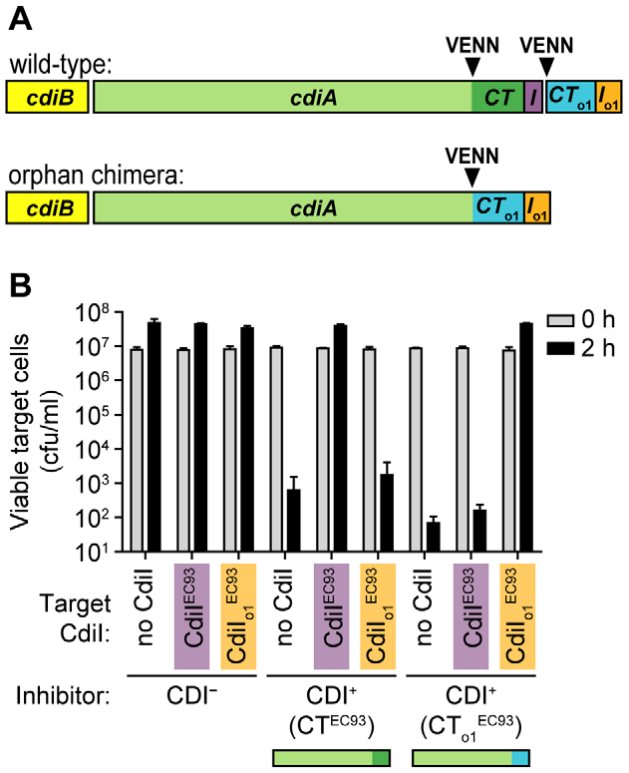
The EC93 orphan *cdiA-CT/cdiI* module is functional in contact-dependent growth inhibition (CDI). A) The wild-type EC93 and orphan chimera CDI systems are shown schematically. The *cdiA-CT* ^EC93^ */cdiI* ^EC93^ region was deleted and orphan module fused onto the *cdiA* ^EC93^ gene at the VENN encoding sequence. B) Growth competitions. CDI^+^ inhibitor cells were co-cultured with target cells expressing either CdiI^EC93^ or orphan CdiI_o1_^EC93^ immunity proteins. Viable target cells were quantified by plating on selective media to determine the number of colony forming units (cfu) per milliliter.

The EC93 orphan *cdiA-CT*_o1_^EC93^ lacks translation initiation signals, suggesting the encoded protein is not synthesized under normal conditions. By analogy with other two-partner secretion proteins, full-length CdiA proteins are secreted through the inner membrane via the general secretory pathway and assembled onto the cell surface through interactions with CdiB [4], [18]. The EC93 orphan *cdiA-CT*_o1_^EC93^ gene also lacks a signal sequence and thus would not be delivered to the cell surface if it were expressed. Therefore to test whether CdiA-CT_o1_^EC93^ and CdiI_o1_^EC93^ are functional in the context of cell-mediated CDI, we replaced the EC93 *cdiA-CT* ^EC93^ */cdiI* ^EC93^ region with the corresponding sequences from the EC93 orphan module. The resulting construct produces a chimeric molecule in which the CdiA-CT_o1_^EC93^ is fused to CdiA^EC93^ at the VENN motif (Figure 6A). *E. coli* expressing the CdiA^EC93^-CT_o1_^EC93^ chimera inhibited the growth of target cells expressing the CdiI^EC93^ immunity protein ∼10^5^-fold compared to control CDI^−^ inhibitor cells, but target cells expressing the orphan CdiI_o1_^EC93^ were completely protected from growth inhibition (Figure 6B). In contrast, CdiI_o1_^EC93^ was unable to protect target cells from growth inhibition mediated by CdiA^EC93^ (Figure 6B). Thus, the EC93 orphan CdiA-CT_o1_ is functional in contact-dependent growth inhibition when it is part of a full-length CdiA protein.

### The Orphan CdiI Immunity Protein Is Expressed in EC93

**Figure 7:**
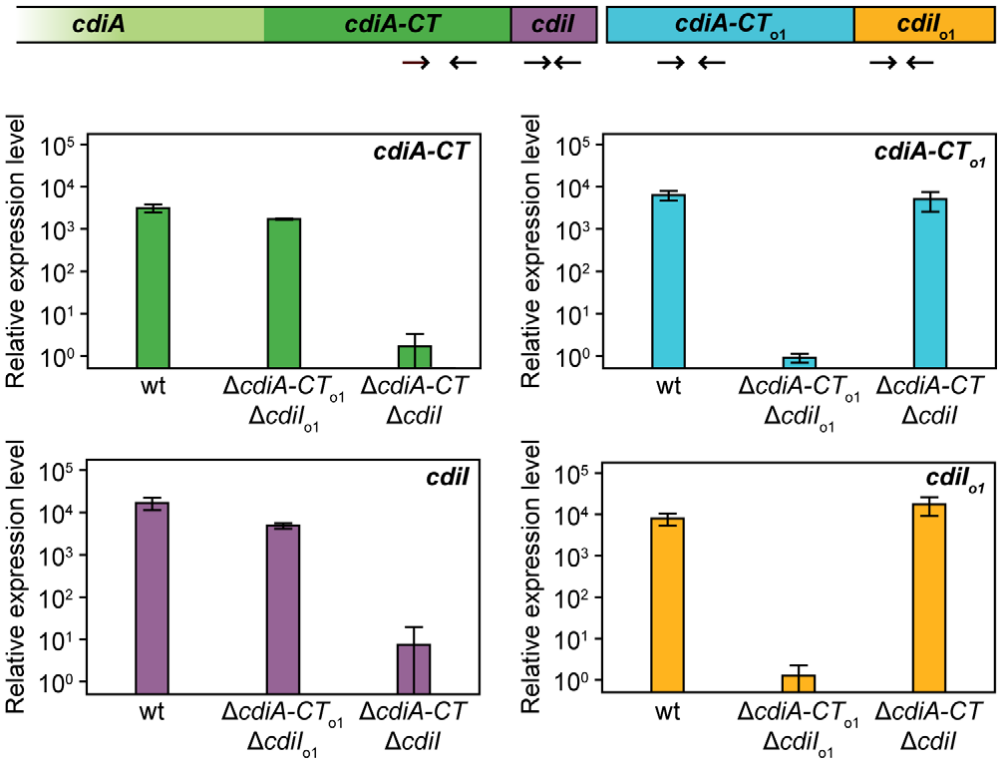
The EC93 orphan region is transcribed. RNA from *E. coli* EC93, EC93 Δ*cdiA-CT* ^EC93^Δ*cdiI* ^EC93^, and EC93 Δ*cdiA-CT*_o1_^EC93^Δ*cdiI*_o1_^EC93^ was subjected to quantitative RT-PCR. The primer binding sites within the *cdi* locus are depicted schematically as arrows. The relative expression levels represent the mean ± SEM for three independently isolated RNA samples.

To determine whether orphan *cdiA-CT/cdiI* modules are expressed, we isolated total RNA from *E. coli* EC93 and performed quantitative RT-PCR using primer pairs to amplify from potential *cdiA-CT* ^EC93^, *cdiI* ^EC93^, *cdiA-CT*_o1_^EC93^ and *cdiI*_o1_^EC93^ transcripts (Figure 7). This analysis revealed that transcripts encoding orphan *cdiA-CT*_o1_^EC93^ and *cdiI*_o1_^EC93^ are expressed in wild-type EC93 cells, and that orphan message levels are very similar to those encoding the main *cdiA-CT* ^EC93^ and *cdiI* ^EC93^ (Figure 7). Additionally, the orphan transcript was expressed at approximately the same level in an EC93 strain deleted for the main *cdiA-CT* ^EC93^/*cdiI* ^EC93^ region (Figure 7), indicating that the promoter driving orphan region transcription is upstream of the main *cdiA-CT* ^EC93^. These results suggest that the orphan region is co-transcribed with the upstream *cdiA* and *cdiI* genes. Indeed, a large RT-PCR product was obtained with the forward *cdiA* ^EC93^ and reverse *cdiA-CT*_o1_^EC93^ primers (data not shown), confirming that all of these ORFs are present on a single transcript. We next sought to detect the CdiA-CT_o1_^EC93^ protein by immunoblot using polyclonal antibodies raised against the homologous CdiA-CT^UPEC536^ from UPEC 536. Although this antiserum detects CdiA-CT_o1_^EC93^ produced from a heterologous expression system in *E. coli* K-12, we were unable to detect CdiA-CT_o1_^EC93^ in whole-cell lysates of EC93 (data not shown).

**Figure 8:**
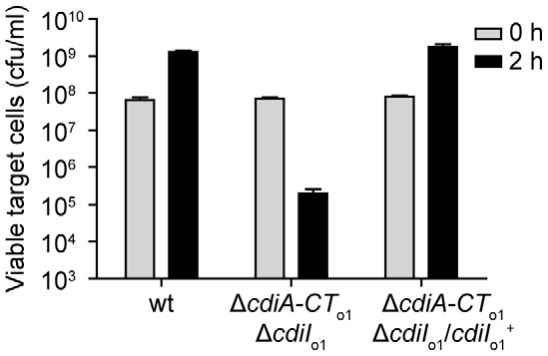
The EC93 orphan region produces functional CdiI_o1_ immunity protein. EC93 expressing chimeric CdiA^EC93^-CT_o1_^EC93^ was used as an inhibitor strain in growth competition experiments. Inhibitor cells were co-cultured with wild-type EC93, EC93 deleted for the orphan region (Δ*cdiA-CT*_o1_^EC93^Δ*cdiI*_o1_^EC93^), and EC93 Δ*cdiA-CT*_o1_^EC93^Δ*cdiI*_o1_^EC93^ cells complemented with a plasmid-borne copy of *cdiI*_o1_^EC93^ (*cdiI*_o1_^+^). Viable target cells were quantified by plating on selective media to determine the number of colony forming units (cfu) per milliliter.

In contrast to the orphan *cdiA-CT*_o1_^EC93^ fragment, the EC93 *cdiI*_o1_^EC93^ gene has an initiation codon and a properly spaced Shine-Dalgarno sequence, indicating that the CdiI_o1_^EC93^ protein is likely to be synthesized in EC93 cells. To determine if orphan CdiI_o1_^EC93^ is indeed expressed, we tested whether EC93 cells are resistant to CDI mediated by the chimeric CdiA^EC93^-CT_o1_^EC93^ protein. Because EC93 cells are CDI^+^, the chimeric inhibitor strain must itself be immune to EC93-mediated CDI. Therefore, we introduced a cosmid encoding the CdiA^EC93^-CT_o1_^EC93^ chimera into EC93 and used the resulting strain as the inhibitor for these experiments. Wild-type EC93 was not inhibited by EC93 expressing the CdiA^EC93^-CT_o1_^EC93^ chimera, but the EC93 Δ*cdiA-CT*_o1_^EC93^/Δ*cdiI*_o1_^EC93^ strain was inhibited approximately 10^3^-fold (Figure 8). This growth inhibition was completely abrogated when orphan CdiI_o1_^EC93^ immunity protein was expressed from a plasmid in the EC93 Δ*cdiA-CT*_o1_^EC93^/Δ*cdiI*_o1_^EC93^ cells (Figure 8). Taken together, these results demonstrate that functional CdiI_o1_^EC93^ immunity protein is produced from the orphan locus in EC93.

### *rhs* Loci Encode Orphan Toxin/Immunity Modules

**Figure 9:**
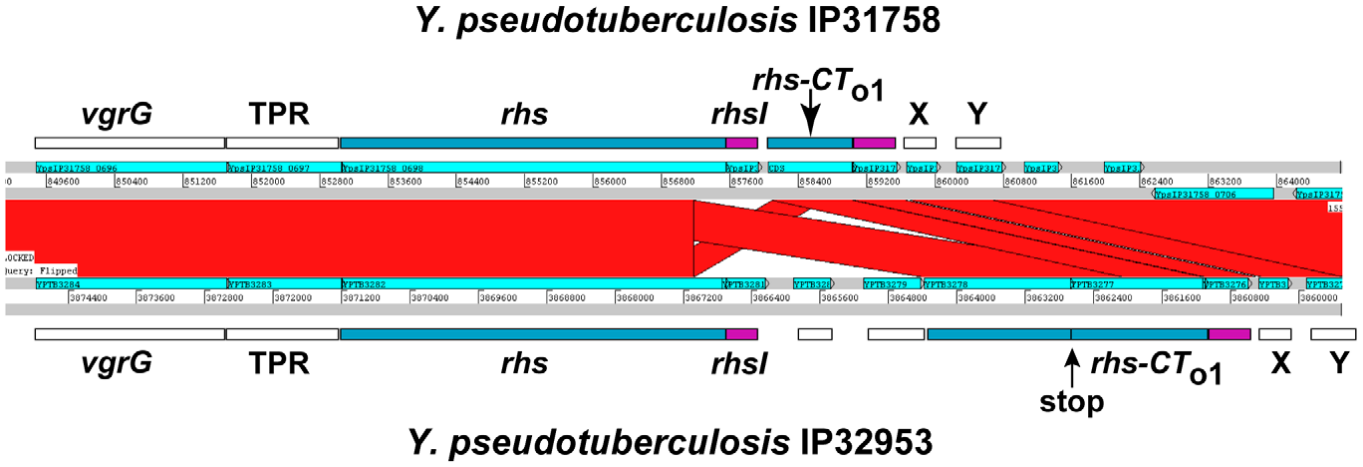
Rhs genes have variable CT encoding sequences, immunity genes, and orphan *rhs-CT/rhsI* modules. ACT comparison of related Rhs regions from *Y. pseudotuberculosis* strains IP31758 (top) and IP32953 (bottom). This region is highly conserved between the two strains, but the *rhs* genes encode unrelated CT sequences and the adjacent *rhsI* genes are unrelated. Both strains contain a related orphan *rhs-CT/rhsI* module. The orphan module in strain IP32953 has more upstream *rhs* coding sequence, but contains an in-frame stop codon in this retained sequence. The *vgrG* gene is a conserved component of Type VI secretion systems; TPR is a conserved gene encoding a potential tetratricopeptide repeat protein; and X and Y are conserved predicted genes of unknown function.

In the course of our bioinformatic analyses, we found that many CdiA-CT sequences share significant identity with the C-terminal regions of Rhs proteins (Table S1). For example, the CdiA-CT^91001(I)^ of *Y. pestis* Microtus strain 91001 (Q74T84) is similar to the C-terminal region of an Rhs/YD-repeat protein from *Waddlia chondrophila* WSU 86–1044 (D6YTT8) (Table S1 and Figure S7). The *W. chondrophila rhs* gene is followed by a small ORF that encodes a protein with similarity to CdiI^91001(I)^ from *Y. pestis* Microtus 91001 (27% identity over 48 residues), suggesting this locus encodes a toxin/immunity protein pair. Intriguingly, Rhs-CT sequences are variable and demarcated by a well-conserved peptide motif (PxxxxDPxGL) analogous to the VENN motif in CdiA proteins [12], [19]. The parallels between CDI and Rhs systems extend to their genetic organization, with many *rhs* loci containing numerous “silent cassettes” that resemble CDI orphan modules [12]. Rhs systems also appear to undergo complex rearrangements that diversify Rhs-CT sequences. For example, in one Rhs region shared by *Y. pseudotuberculosis* strains IP31758 and IP32953, the *rhs-CT* and putative *rhsI* sequences are completely unrelated to one another, but the surrounding genomic regions are clearly homologous between the strains (Figure 9). This homology includes *rhs* coding sequence upstream of the region encoding DPxGL and nearly identical *rhs-CT/rhsI* orphan modules downstream of the *rhs* genes (Figure 9 and Figure S8). In addition, orphan *rhs-CT/rhsI* modules from a given species are often found fused to full-length *rhs* genes in other bacteria. For example, one of the *rhs* loci in *D. dadantii* 3937 contains two orphan *rhs-CT/rhsI* modules. Both of these predicted orphan Rhs-CT^3937^ proteins are related to the C-terminal regions of full-length Rhs proteins: Rhs-CT_o1_^3937^ (E0SGM0) is related to the CT region of a putative Rhs repeat protein (C3K5K6; 49% identity in 147 residues following the DPxGL) from *Pseudomonas fluorescens* SBW25, and Rhs-CT_o2_^3937^ (E0SGM2) is related to the CT of a predicted YD-repeat protein (C6CNW6; 99% identity in 116 residues) from *Dickeya zeae* 1591.

**Figure 10:**
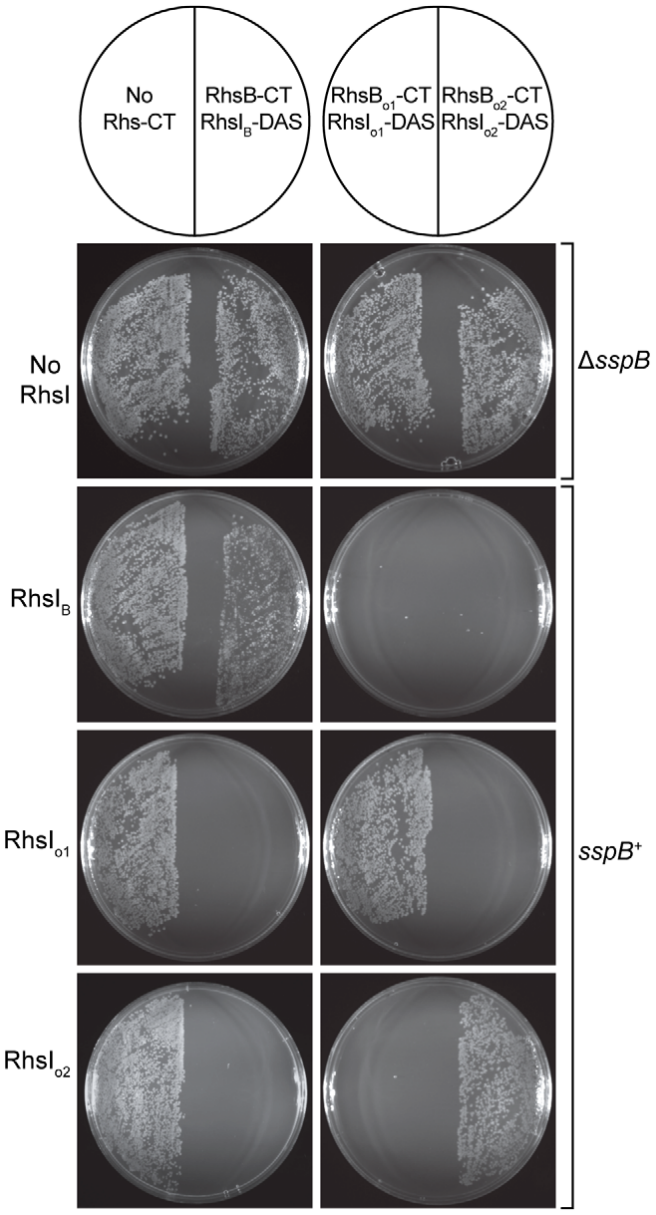
The Rhs genes of *D. dadantii* 3937 encode toxin/immunity pairs. *E. coli sspB* ^+^ cells expressing RhsI^3937^ proteins were incubated with supercoiled plasmids encoding the various Rhs-CT^3937^/RhsI^3937^-DAS pairs and plated to select stable transformants. All Rhs-CT^3937^/RhsI^3937^-DAS constructs were also introduced into *E. coli* Δ*sspB* cells to demonstrate that RhsI^3937^-DAS degradation is required for growth inhibition.

In general, the functions of Rhs proteins are unknown, but data from Hill and colleagues suggest that that the *E. coli rhsA* locus may encode a toxin/immunity pair [20]. In conjunction with the similarities to CDI systems, these observations suggest that Rhs proteins may be involved in intercellular competition. To test whether other Rhs systems encode toxin/immunity pairs, we examined *rhs* genes from *D. dadantii* 3937, which contains three full-length *rhs* genes that we have termed *rhsA* (Dda3937_01758), *rhsB* (Dda3937_02773) and *rhsC* (Dda3937_04312). Each of these *rhs* genes is closely followed by a small ORF that encodes a possible immunity protein. Additionally, the *rhsC* locus contains the two orphan *rhs-CT* gene fragments described above. We first tested RhsB-CT^3937^ for growth inhibitory function, because this domain contains an HNH endonuclease motif found in other cytotoxic proteins [21]. We expressed RhsB-CT^3937^ together with an ssrA(DAS)-tagged version of the putative RhsI_B_^3937^ immunity protein in *E. coli* cells, and found that growth was arrested upon degradation of RhsI_B_^3937^-(DAS) (Figure 10 and data not shown). The same results were obtained with the two orphan modules from *D. dadantii* 3937 (Figure 10 and data not shown), indicating that Rhs-CT_o1_^3937^ and Rhs-CT_o2_^3937^ also have growth inhibition activity. These results also demonstrate that the putative *rhsI* genes do in fact encode proteins with immunity function.

Finally, we examined the specificity of RhsI-mediated immunity. Although each Rhs-CT/RhsI-(DAS) expression construct can be maintained stably in *E. coli* Δ*sspB* strains, these plasmids cannot be transformed into *sspB* ^+^ cells due to the Rhs-CT toxicity that results from RhsI-(DAS) degradation (Figure 10 and data not shown). Therefore, we asked whether untagged RhsI protein could rescue cells from Rhs-CT toxicity and allow stable transformation of Rhs-CT/RhsI-(DAS) plasmids into *sspB^+^* cells. Each of the three Rhs-CT/RhsI-(DAS) plasmids were introduced into *sspB* ^+^ cells expressing individual RhsI proteins, and the transformed cell suspensions plated onto selective media containing L-arabinose to induce Rhs-CT/RhsI-(DAS) synthesis. Transformants carrying the Rhs-CT/RhsI-(DAS) expression plasmid were only obtained if the cells also expressed the cognate RhsI immunity protein (Figure 10). Therefore, each RhsI protein only confers immunity to its cognate Rhs-CT, demonstrating that the Rhs systems of *D. dadantii* 3937 encode polymorphic toxin/immunity pairs.

## Discussion

The results presented here show that many CDI systems have a complex genetic organization in which the *cdiBAI* genes are followed by an array of orphan *cdiA-CT/cdiI* modules. These arrays can be extensive, with eleven orphan gene pairs in the *cdi* locus of *E. coli* EC869. The widespread occurrence of orphan *cdiA-CT/cdiI* modules in diverse bacterial species argues that these gene pairs confer a selective advantage. Although the majority of orphan *cdiA-CT* genes lack translation initiation signals, orphan *cdiI* genes appear to be fully capable of expression. Therefore, bacteria could maintain orphan modules to build a repertoire of immunity genes to protect themselves from neighboring bacteria that express diverse CdiA proteins. Broad range immunity would clearly confer an advantage and our data demonstrate that EC93 does in fact express its orphan CdiI immunity protein. However, if bacteria are collecting immunity genes, then it is unclear why the toxin-encoding *cdiA-CT* fragments are retained in the process. One model to explain these findings is that orphan modules allow the bacterium to change its toxin/immunity profile by recombination between the highly conserved VENN-encoding sequences. This may have occurred in the *cdi* locus of *Y. pestis* Microtus 91001, where a large deletion has loaded an orphan *cdiA-CT/cdiI* module onto the main *cdiA* gene. However, such a strategy for generating CdiA-CT diversity could have serious shortcomings. First, although reloading CdiA with a new toxic C-terminal domain would produce a novel weapon, the recombination would also delete *cdiI* and render the cell susceptible to the original CdiA protein expressed by neighboring bacteria that have not recombined their *cdi* locus. Second, simple recombination between *cdiA* and distal orphan *cdiA-CT* genes would delete all intervening *cdiA-CT/cdiI* modules. An alternative model is that recombination between *cdiA* (or *rhs*) and the orphan regions occurs following tandem duplication of the loci. This mechanism would allow *cdiA-CT/cdiI* modules to be rearranged without the loss of immunity or genetic diversity, because a copy of the original *cdi/rhs* system would be present [22], [23].

The results presented here have also revealed a connection between CDI and Rhs systems. The *rhs* genes were first described in *E. coli*, and were originally thought to be rearrangement hot spots because of a recombination event between nearly identical sequences within the *rhsA* and *rhsB* genes [10], [11]. These proteins are widely distributed throughout the eubacteria and related proteins containing YD peptide repeats are also found in metazoans including all vertebrates. Despite their prevalence, the function of these proteins is almost completely unknown. Our data show that at least some Rhs systems encode toxin/immunity protein pairs, and a recent bioinformatic study has proposed that Rhs proteins contain toxic nuclease domains [24]. These results are consistent with work from Hill and colleagues showing that the C-terminal region of *E. coli* RhsA blocks the recovery of stationary phase cells [20]. Moreover, this growth inhibition was neutralized by expression of the ORF (*yibA*) encoded immediately downstream of *rhsA* [20]. More recently an Rhs-related protein from *Pseudomonas savastanoi* pv. savastanoi was found to be associated with bacteriocin activity [25]. Because of the parallels between CDI and Rhs, we suspect that Rhs proteins are also exported to block the growth of neighboring cells and thereby impart a competitive advantage to the inhibitor cell. However, many Rhs proteins from Gram-negative bacteria lack recognizable signal sequences, so the export pathway is unclear in several instances. Many *rhs* genes are linked to valine-glycine repeat (Vgr)-encoding genes that are associated with Type VI secretion systems [26]. VgrG proteins associated with Type VI secretion systems have structural similarity to bacteriophage cell-puncturing proteins [27], [28], and are therefore ideal for penetrating bacterial envelopes. Indeed, recent work has demonstrated that Type VI systems are used to deliver protein toxins into target bacteria [29]. Whether the close genetic linkage between *rhs* and *vgr* genes extends to a functional relationship remains to be determined.

Although the Rhs systems examined here appear to be growth inhibitory, there are indications that Rhs proteins have other signaling modalities. Youdarian and Hartzell found that an Rhs-related YD repeat protein (Q1CXS7) from *Myxococcus xanthus* plays an important role in social motility [15], which occurs when individual bacteria make contact with one another to coordinate cell movement. The *M. xanthus* YD repeat protein has a signal sequence and is presumably exported to the surface. The gene encoding Q1CXS7 (MXAN_6679) is closely followed by a small ORF that is suggestive of an *rhsI* gene. However, it is not clear that immunity would be required in this signaling pathway. Perhaps this small ORF encodes a peptide that is involved in receiving the signal from the C-terminal region of the YD repeat protein. Recent work on the entomopathogen *Pantoea stewartii* suggests another function for a class of Rhs-related proteins termed Ucp (for you cannot pass) [30]. The *P. stewartii* genome contains seven *ucp* homologs that encode large proteins (1,200–1,300 residues) with similar N-terminal regions and variable C-terminal sequences. The Ucp1 protein mediates bacterial aggregation and is a virulence factor required for pathogenicity against the aphid host. It was postulated that Ucp1 and other Ucp proteins function primarily as adhesins and that C-terminal variability is driven by the need to evade host immune responses [30]. Our results suggest an alternative possibility. The C-terminal peptide of Ucp1 could be delivered into insect cells and exert a toxic effect in a manner similar to that proposed for CDI [5]. In this model, the other six Ucp proteins could be targeted against other bacterial competitors or different eukaryotic hosts.

Finally, another intriguing example of Rhs signaling has been proposed for teneurins. The teneurin protein family is comprised of four paralogs that are present in all vertebrates [31]. These proteins are type II integral membrane proteins (single transmembrane span with the C-terminus presented extracellularly) that play a role in axon guidance and neural patterning during development [31], [32]. Like Rhs proteins, the teneurins are large (2,500–2,800 residues) and the C-terminal half is comprised of several YD peptide repeats. Remarkably, all teneurins contain a C-terminal associated peptide (TCAP) that is similar to neuroendocrine signaling peptides [33], [34]. The TCAP region is adjacent to a phylogenetically conserved furin protease cleavage site (RxRR) [31], suggesting that the TCAPs are released and enter target cells to exert neuromodulatory effects. Because some CdiA-CTs and Rhs-CTs have toxic nuclease activity, presumably they must also be cleaved for delivery into the cytoplasm of target cells. The parallel between CDI/Rhs-mediated growth inhibition and the proposed teneurin signaling pathway is striking, suggesting that intercellular communication through the delivery of cleaved C-terminal peptides is ubiquitous and possibly ancient. Perhaps these systems have arisen from a common YD-repeat protein ancestor.

## Materials and Methods

### Bioinformatic Analysis

Pairwise sequence comparisons were performed using GCG Gap or pairwise BLAST. Related CdiA-CT, Rhs-CT, CdiI, RhsI and orphan sequences were found using TBLASTN against assembled bacterial genomes, because these regions are often not annotated and thus not represented in the non-redundant protein database. Pairwise comparisons of genomic regions were performed using WebACT (www.webact.org) with BLASTN comparisons. The UniProt, GenBank and gene locus accession numbers for each full-length CdiA and Rhs protein discussed in this work are presented in Table S2.

### Plasmid Constructs

The orphan *cdiA-CT*_o1_^EC93^ */cdiI*_o1_^EC93^ module from *E. coli* EC93 was amplified using oligonucleotides, **EC93orph-Nco** (5′ - TTG CCA TGG AGA ATA ACT CGC TGA GC) and **EC93orph-Spe** (5′ - ATC ACT AGT GGC ATT AGA TAG CTT ATC TAT TTT TGC) (restriction endonuclease sites are underlined), followed by digesting with NcoI and SpeI, and ligation to plasmid pET21S [5]. The resulting construct overproduces CdiA-CT_o1_^EC93^ and C-terminally His_6_-tagged CdiI_o1_^EC93^. The EC93 orphan immunity gene was amplified using primers **#1658** (5′ - CAA CAA GCA TGC CCC GAC TTT GAG ACC AGA ATA TC) and **#1664** (5′ - ATC AGG AGC ATG GTA TAT GAC AAC ATT TAG ATC). The resulting PCR product was digested with SphI and ligated to EcoRV/SphI digested plasmid pBR322 under control of the *tet* promoter.

Orphan *cdiA-CT/cdiI* modules from *D. dadantii* 3937 and *E. coli* EC869 were amplified using **3937orph-Nco** (5′ - AAG CCA TGG TGG AGA ATA ACT ATC TGA GCA G) and **3937orph-Spe** (5′ - TCT ACT AGT AGG CTG GTA ATC TTC ATA TTC C); and **EC869orph11-Nco** (5′ - ATT CCA TGG GCA CAA ACC AGT CTC TGA CCT TCG) and **EC869orph11-Spe** (5′ - TCT ACT AGT ACC TTT GCA GCG ACT CAA GGC CAG), respectively. The *rhs-CT* ^3937^ */rhsI* ^3937^ modules from *D. dadantii* 3937 were amplified using the following primer pairs: **rhsB-Nco** (5′ - CAG CCA TGG AAA GTA ATT ACG GTT ATG TCC) and **rhsB-Spe** (5′ -AAA CTA GTA ATT TTT CTT GAT TTA TAT TTT ACA AGC); **rhs-orph1-Nco** (5′ - TCC CAT GGG GTT GGT GGG ATG TCC GC) and **rhs-orph1-Spe** (5′ - AAA ACT AGT GCC ATC AAG GTA TAC AGA AGG); and **rhs-orph2-Nco** (5′ - ACC CCA TGG GGC TGG CAG GGG GGC TG) and **rhs-orph2-Spe** (5′ - TTT ACT AGT AAC AGC TTT GTA ATA ATC GTG). All PCR products were digested with NcoI and SpeI and ligated to pET21S. The *D. dadantii rhsI* ^3937^ genes were amplified from pET21S constructs using primer **pET-Pst** (5′ - CGG CTG CAG CAG CCA ACT CAG TGG) in conjunction with primers: **rhsI_B_-Nco** (5′ - TAA CCA TGG ATA TTG AAA ATG C), **rhsI_o1_-Nco** (5′ - TCA CCA TGG ATT CTA GTG ATA AG), and **rhsI_o2_-Nco** (5′ - AAT CCA TGG ATG CTG AAC AAT TTG). The resulting PCR products were digested with NcoI and PstI and ligated to plasmid pCH450 [35].

A cassette encoding the ssrA(DAS) peptide tag (AANDENYSENYADAS) was generated from oligonucleotides, **DAS-top** (5′ - CTA GTG CTG CGA ACG ATG AAA ATT ACT CCG AAA ATT ATG CGG ATG CGT CTT AAT G) and **DAS-bot** (5′ - GAT CCA TTA AGA CGC ATC CGC ATA ATT TTC GGA GTA ATT TTC ATC GTT CGC AGC A), and ligated to SpeI/BamHI digested plasmid pKAN [36]. As part of an unrelated study, a fragment of the *E. coli hisS* gene was cloned into pKAN(DAS) using SacI and SpeI sites. The resulting *hisS-(DAS)* SacI/BamHI fragment was subcloned into plasmid pTrc99A (Amersham Pharmacia) to generate plasmid pTrc(DAS). All *cdiA-CT/cdiI* and *rhs-CT/rhsI* modules were subcloned into pTrc(DAS) using NcoI and SpeI restriction sites. The *sspB* and *sspB(Δ47)* genes were amplified using **SspB-Nde** (5′ - GAG TTA ATC CAT ATG GAT TTG TCA CAG C) in combination with **SspBΔ47-Bam** (5′ - TGC GGA TCC TTA ATT CAT GAT GCT GGT ATG TTC ATC GTA GGC) and **SspB-Bam** (5′ - ATA TGA TTG CCA GGA TCC CGC TAT TTT ATT AAG TC), respectively. Both PCR products were digested with NdeI and BamHI and ligated to plasmid pCH410, allowing L-arabinose control of SspB and SspB(Δ47) expression [37].

The orphan *cdiA-CT*_o1_^EC93^ */cdiI*_o1_^EC93^ module from *E. coli* EC93 was fused to the full-length EC93 *cdiA* ^EC93^ gene in multiple steps. A fragment of *cdiA* ^EC93^ (upstream of the VENN encoding sequence) was amplified using primers, **#1527** (5′ - GAA CAT CCT GGC ATG AGC G) and **#1758** (5′ - CAA GCT CAG CGA GTT ATT CTC AAC CGA GTT CCT ACC TGC CTG). The EC93 orphan module was amplified using primers, **#1759** (5′ - CAG GCA GGT AGG AAC TCG GTT GAG AAT AAC TCG CTG AGC TTG) and **#1663** (5′ - GGT CTG GTG TCT AAC CTT TGG G). The two products were combined by overlapping-end PCR using primers **#1527** and **#1663**, and the resulting product digested with SphI and AvrII and ligated to plasmid pDAL660Δ1-39 [4].

### *In Vitro* Characterization of Orphan Modules

All CdiA-CT/CdiI-His_6_ complexes were overproduced and purified by Ni^2+^-affinity chromatography as described [5]. Complexes were eluted from Ni^2+^-nitrilotriacetic acid resin with native elution buffer [20 mM sodium phosphate (pH 7.0) –10 mM β-mercaptoethanol – 250 mM imidazole], followed by dialysis in storage buffer [20 mM sodium phosphate (pH 7.0) – 150 mM NaCl – 10 mM β-mercaptoethanol]. CdiA-CT and CdiI-His_6_ proteins were separated from one another by Ni^2+^-affinity chromatography with denaturing buffer [20 mM sodium phosphate (pH 7.0) – 10 mM β-mercaptoethanol – 6 M guanidine-HCl]. Denatured proteins were refolded by dialysis into storage buffer. All purified proteins were quantified by absorbance at 260 nm using the following molar extinction coefficients: CdiA-CT^UPEC536^, 12,950 M^−1^ cm^−1^; CdiI^UPEC536^, 8,480 M^−1^ cm^−1^; CdiA-CT_o1_^EC93^, 11,460 M^−1^ cm^−1^; and CdiI_o1_^EC93^, 11,460 M^−1^ cm^−1^. The specificity of CdiA-CT/CdiI-His_6_ binding interactions was determined by Ni^2+^-affinity co-purification as described [5]. The tRNase activity of isolated and refolded CdiA-CT^UPEC536^ and CdiA-CT_o1_^EC93^ proteins was determined as described [5], [37].

### *In Vivo* Activity of Orphan Modules

*E. coli* strain CH4180 (×90 Δ*sspB*) was co-transformed with pTrc(DAS) orphan module constructs and either the SspB or SspB(Δ47) arabinose-inducible expression plasmids. The resulting strains were grown at 37°C with aeration in LB media supplemented with 150 µg/mL ampicillin and 10 µg/mL tetracycline (to maintain plasmids) to mid-log phase and re-diluted into fresh media to an optical density at 600 nm (OD_600_) of 0.05. After 40 min, SspB or SspB(Δ47) expression was induced by the addition of 0.4% L-arabinose. Cell growth was tracked by measuring the OD_600_ every 30 min after induction. Cells expressing CdiA-CT_o1_^EC93^/CdiI_o1_^EC93^-DAS were harvested into an equal volume of ice-cold methanol and RNA extracted as described [38]. Northern blot hybridizations were conducted as described [37], using radiolabeled oligonucleotides **tRNA^His^ probe** (5′ - CAC GAC AAC TGG AAT CAC) and **tRNA_1B_^Ala^ probe** (5′ - TCC TGC GTG AGC AG) as probes.

*E. coli sspB* ^+^ cells expressing RhsI_B_^3937^, RhsI_o1_^3937^ and RhsI_o2_^3937^ were transformed with plasmids encoding *rhs-CT/rhsI(DAS)* modules under control of the P_BAD_ promoter [39]. Competent cells were incubated with 0.5 µg of purified supercoiled plasmid for 20 min on ice, then heat-shocked at 42°C for 45 s. The treated cells were recovered in 1.0 mL of LB media for 2 hr without selection, then 20 µL of the cell suspension was plated onto LB-agar supplemented with ampicillin (150 µg/mL), tetracycline (10 µg/mL) and 0.4% L-arabinose. *E. coli* Δ*sspB* cells were also transformed in the same manner with arabinose-inducible *rhs-CT/rhsI(DAS)* constructs to confirm that growth inhibition was dependent upon RhsI-DAS degradation.

### EC93 *cdiA-CT/cdiI* Deletions

The main *cdiA-CT* ^EC93^ region and *cdiI* ^EC93^ gene were deleted from rifampicin-resistant *E. coli* strain EC93 (DL3852) using allelic exchange as described [4], [40]. Sequence upstream of *cdiA-CT* ^EC93^ was amplified using oligonucleotides **#1683** (5′ - CAA CAA GAG CTC GAA CAT CCT GGC ATG AGC G) and **#1684** (5′ - CAG CGA GTT ATT CTC AAC AAC AAC TA CGA GTT CCT ACC TGC CTG) (SacI restriction endonuclease site is underlined). Sequence downstream of *cdiI* ^EC93^ (including the *cdiI* ^EC93^ stop codon) was amplified using oligonucleotides **#1685** (5′ - CAG GCA GGT AGG AAC TCG tag TTG TTG TTG AGA ATA ACT CGC TG) and **#1686** (5′ - CAA CAA TCT AGA CCC GAC TTT GAG ACC AGA ATA TC) (XbaI restriction endonuclease site is underlined). The two PCR products were combined by overlapping-end PCR using primers **#1683** and **#1686**, and the resulting product digested with SacI and XbaI and ligated to suicide vector pRE112 [40].

The orphan *cdiA-CT*_o_^EC93^/*cdiI*_o_ *^EC93^* module was deleted from rifampicin-resistant EC93 in a similar manner. Sequence upstream of *cdiA-CT*_o1_^EC93^ was amplified using oligonucleotides **#1714** (5′ - CAA CAA GAG CTC GTG AAG GTG GGC TTA CTC AG) and **#1715** (5′ - CGA CTT TGA GAC CAG AAT ATC TAT TTA CTC AAC AAC AAC TAT TTT CTG TCT AAG) (SacI restriction endonuclease site is underlined). Sequence downstream of *cdiI*_o1_^EC93^ (including the *cdiI*_o1_^EC93^ stop codon) was amplified using oligonucleotides **#1716** (5′- CTT AGA CAG AAA ATA GTT GTT GTT GAG TAA ATA GAT ATT CTG GTC TCA AAG TCG) and **#1717** (5′ - CAA CAA TCT AGA CCC GTA AGT ATG CTT ATC CCA TG) (XbaI restriction endonuclease site is underlined). The two PCR products were combined by overlapping-end PCR using primers **#1714** and **#1717**, and the resulting product digested with SacI and XbaI and ligated to suicide vector pRE112 [40].

### Growth Competition Assays

*E. coli* strain EPI100 carrying plasmids pWEB-TNC (CDI^−^), pDAL660Δ1-39 (CdiA^EC93^), or pDAL879 (CdiA^EC93^-CT_o1_^EC93^ chimera) were grown overnight at 37°C in LB media supplemented 100 µg/mL of ampicillin. Overnight cultures were diluted into fresh medium to OD_600_ of 0.05, and incubated at 37°C with aeration until the culture reached mid-log phase (OD_600_≈0.3). The log-phase inhibitor cultures were then mixed with target *E. coli* cells – CAG18439 pBR322 (no CdiI), pDAL741 (CdiI^EC93^), or pDAL867 (CdiI_o1_^EC93^) – at an inhibitor to target cell ratio of 10∶1. The co-cultures were incubated for 2 hr at 37°C with aeration. Viable target cell counts were determined by serially dilution of the co-cultures into M9 salt solution followed by plating onto LB agar supplemented with 10 µg/mL of tetracycline.

To assay CdiI_o1_ expression in EC93, growth competitions were conducted with streptomycin-resistant EC93 (DL6104) carrying pDAL879 (CdiA^EC93^-CT_o1_^EC93^ chimera) as the inhibitor strain. Target strains were rifampicin-resistant EC93 carrying plasmid pBR322 (no CdiI), and rifampicin-resistant EC93 Δ*cdiA-CT*_o1_ Δ*cdiI*_o1_ cells carrying pBR322 (no CdiI) or pDAL867 (CdiI_o1_^EC93^). Growth competitions were conducted as described above except that mid-log phase cells were mixed and co-cultured at an inhibitor to target ratio of 1∶1. Viable target cell counts were determined by serial dilution of the co-cultures into M9 salt solution followed by plating onto LB agar supplemented with 150 µg/mL of rifampicin.

### Quantitative RT-PCR Analysis

Total RNA was isolated from wild-type EC93 and EC93 strains deleted for *cdiA-CT* ^EC93^/*cdiI* ^EC93^ and *cdiA-CT*_o1_^EC93^/*cdiI*_o1_^EC93^, followed by treatment with RNase-free DNase I (Roche) to remove contaminating chromosomal DNA. RNA (0.5 µg) was reverse transcribed using the iScript cDNA synthesis kit (Bio-Rad). A control without reverse transcriptase was also prepared to assess chromosomal DNA contamination. Quantitative PCR was carried out on a Bio-Rad MyiQ single-color real-time PCR detection system using SYBR green supermix. The following primers sets were used for amplification: **#1700** (5′ - GGT GAA GGT GGG CTT ACT CA) and **#1701** (5′ - TGA TGT GAC AGA GCC AAA GC) for *cdiA-CT* ^EC93^; **#1698** (5′ - TGC TAT GTA CTG TAC TTG GTC) and **#1699** (5′ - TAA AGC CTA TGG GAT TCC T) for *cdiI* ^EC93^; **#1647** (5′ - ACT GAC CGC TGA TGA ACT GG) and **#1648** (5′ - AGT AGC CGC TTG AAC CTG CAC) for *cdiA-CT*_o1_^EC93^; **#1649** (5′ - TGA ACC CAA CAG TCG CTC TTC) and **#1650** (5′ - GTC TTC CCC AGC CAG AGG AT) for *cdiI*_o1_^EC93^; **#1568** (5′ - TCA CCC CAG TCA TGA ATC AC) and **#1569** (5′ - TGC AAC TCG ACT CCA TGA AG) for 16S rRNA. Thermal cycling conditions were: 95°C for 5 min for polymerase activation and collection of experimental well factors and 40 cycles at 95°C for 10 s; 56°C for 30 s and 72°C for 30s followed by a melting curve (55°C to 95°C) to analyze the end product. Data were analyzed using the iQ5 optical system software (Bio-Rad) and exported to Microsoft Excel and Prism 5.0 for further analysis. For each target gene, a standard curve was generated to assess PCR efficiency (E) allowing the expression level (e) to be determined, where (e) = (E_target_)^−Ct target^/(E_ref_)^−Ct ref^ [41]. Gene expression was normalized to a 16S rRNA RT-PCR product amplified from the corresponding sample, and the reported values represent the mean ± SEM from three independent RNA extractions.

## Supporting Information

Figure S1 The EC93 orphan CdiA-CT/CdiI protein pair is related to the CdiA-CT/CdiI proteins from *E. coli* UPEC 536. A) Pairwise alignment of CdiA-CT_o1_^EC93^ and CdiA-CT^UPEC536^ shows 76% sequence identity. Numbering begins at the Val residue of the conserved VENN motif. B) Alignment of CdiI_o1_^EC93^ and CdiI^UPEC536^ shows 35% sequence identity.
(TIF)

Figure S2 Orphan *cdiA-CT* fragments contain varying amounts of *cdiA* sequence upstream of the region encoding VENN. A) The region II *cdi* locus from *Y. pseudotuberculosis* PB1/+. *CT*_o1_ to *CT*_o4_ designate the orphan *cdiA-CT*_o_^PB1(II)^ genes numbered according to their position in the locus. Dots above *cdiA* ^PB1(II)^ represent every 500 amino acids of the encoded protein. B) Pairwise alignments of the full-length *Y. pseudotuberculosis* PB1/+ region II *cdiA* gene with the linked orphan *cdiA-CT* sequences. The region where similarity with the full-length *cdiA* ^PB1(II)^ gene begins is shown for each orphan *cdiA-CT* sequence. Gray shading indicates nucleotide identity, and the numbers correspond to amino acid residues of the full-length CdiA^PB1(II)^ protein. Amino acid sequences are given in one-letter code and asterisks (*) indicate termination codons. C) The nucleotide and predicted amino acid sequences for the VENN-encoding regions are presented. Orphan sequences shaded in gray are identical to that of the full-length *cdiA* ^PB1(II)^ gene.
(TIF)

Figure S3 Multiple sequence alignment of *Y. pseudotuberculosis* PB1/+ *cdiA-CT* nucleotide sequences. The nucleotide sequences encoding orphan *cdiA-CT*s from the *Y. pseudotuberculosis* PB1/+ region II *cdi* locus were aligned to the full-length *cdiA* ^PB1(II)^ gene. Numbering corresponds to the full-length CdiA^PB1(II)^ protein sequence. Black indicates positions of sequence identity with the full *cdiA* ^PB1(II)^ gene. Sequence identity falls off abruptly after the VENN-encoding region (grey).
(PDF)

Figure S4 VENN-encoding sequences demarcate orphan *cdiA-CT* fragments. The orphan *cdiA-CT*_o1_ of *E. coli* 254 is related to orphan *cdiA-CT*_o7_ of *E. coli* EC869. The conserved region is shaded gray, and the left and right junctions of the conserved region are presented in detail. Conservation begins at the VENN-encoding regions for *cdiA-CT*_o1_^254^ and *cdiA-CT*_o7_^EC869^ (left junction) and extends through the predicted orphan *cdiI* genes to the VENN encoding regions of the following orphan *cdiA-CT* genes (99% sequence identity in 1048 nucleotides). The sequences then diverge immediately after VENN encoding regions *cdiA-CT*_o2_^254^ and *cdiA-CT*_o8_^EC869^.
(TIF)

Figure S5 The CdiA_I_-CT of *Y. pestis* Microtus 91001 is unrelated to CdiA-CTs from other *Y. pestis* strains. Pairwise alignment of CdiA^(I)^ proteins encoded by the region I *cdi* loci of *Y. pestis* Microtus 91001 and *Y. pestis* CO92. Numbers correspond to amino acid residues of the full-length predicted CdiA^(I)^ proteins. Regions of sequence identity are shaded gray and the VENN peptide motif is boxed.
(TIF)

Figure S6 Orphan *cdiA-CT/cdiI* modules from *D. dadantii* 3937 and *E. coli* EC869 encode functional toxin/immunity pairs. A) Purification of orphan CdiA-CT/CdiI-His_6_ proteins. CdiA-CT_o1_^3937^/CdiI_o1_^3937^-His_6_ and CdiA-CT_o11_^EC869^/CdiI_o11_^EC869^-His_6_ complexes were purified by Ni^2+^-affinity chromatography under non-denaturing conditions and analyzed by SDS-PAGE. B) Growth curves of *E. coli* Δ*sspB* cells expressing orphan CdiA-CT/CdiI-DAS complexes. Degradation of CdiI-DAS proteins was initiated by the addition of L-arabinose to induce SspB synthesis. Control cells express SspB(Δ47), which does not deliver CdiI-DAS proteins to the ClpXP protease. Growth curves with square symbols represent control strains expressing SspB or SspB(Δ47), but not orphan CdiA-CT/CdiI-DAS complexes.
(TIF)

Figure S7 The CdiA_I_-CT of *Y. pestis* Microtus 91001 is related to an Rhs-CT from *Waddlia chondrophila*. Pairwise alignment of *Y. pestis* Microtus CdiA-CT^91001(I)^ (Q74T84) and the C-terminal region of a predicted Rhs/YD-repeat protein from *Waddlia chondrophila* WSU 86–1044 (D6YTT8). Regions of sequence identity are shaded gray and the PxxxxDPxGL and VENN peptide motifs are boxed.
(TIF)

Figure S8 The C-terminal regions of Rhs proteins are variable. Pairwise alignment of related Rhs proteins from *Y. pseudotuberculosis* IP31758 and *Y. pseudotuberculosis* IP32953. Regions of identity are shaded gray. Sequences diverge abruptly after the DPxGL motif (boxed).
(TIF)

Table S1 Homologies between selected CdiA-CT and Rhs-CT sequences. Rhs-CT sequences were identified by BLAST searches using selected CdiA-CTs as the query sequences. The presence of probable *rhs-CT/rhsI* orphan modules is indicated for each Rhs system.
(XLS)

Table S2 Sequence identifiers for CdiA and Rhs proteins. The UniProt, GenBank and locus tag accession numbers are presented for each CdiA and Rhs protein discussed in the text.
(XLS)
