# Supplementary figures and images for: Identification of Functional Toxin/Immunity Genes Linked to Contact-Dependent Growth Inhibition (CDI) and Rearrangement Hotspot (Rhs) Systems
Source: PLoS Genet. 2011 Aug 4;7(8):e1002217. doi: 10.1371/journal.pgen.1002217 (PMC3150448; doi:10.1371/journal.pgen.1002217)

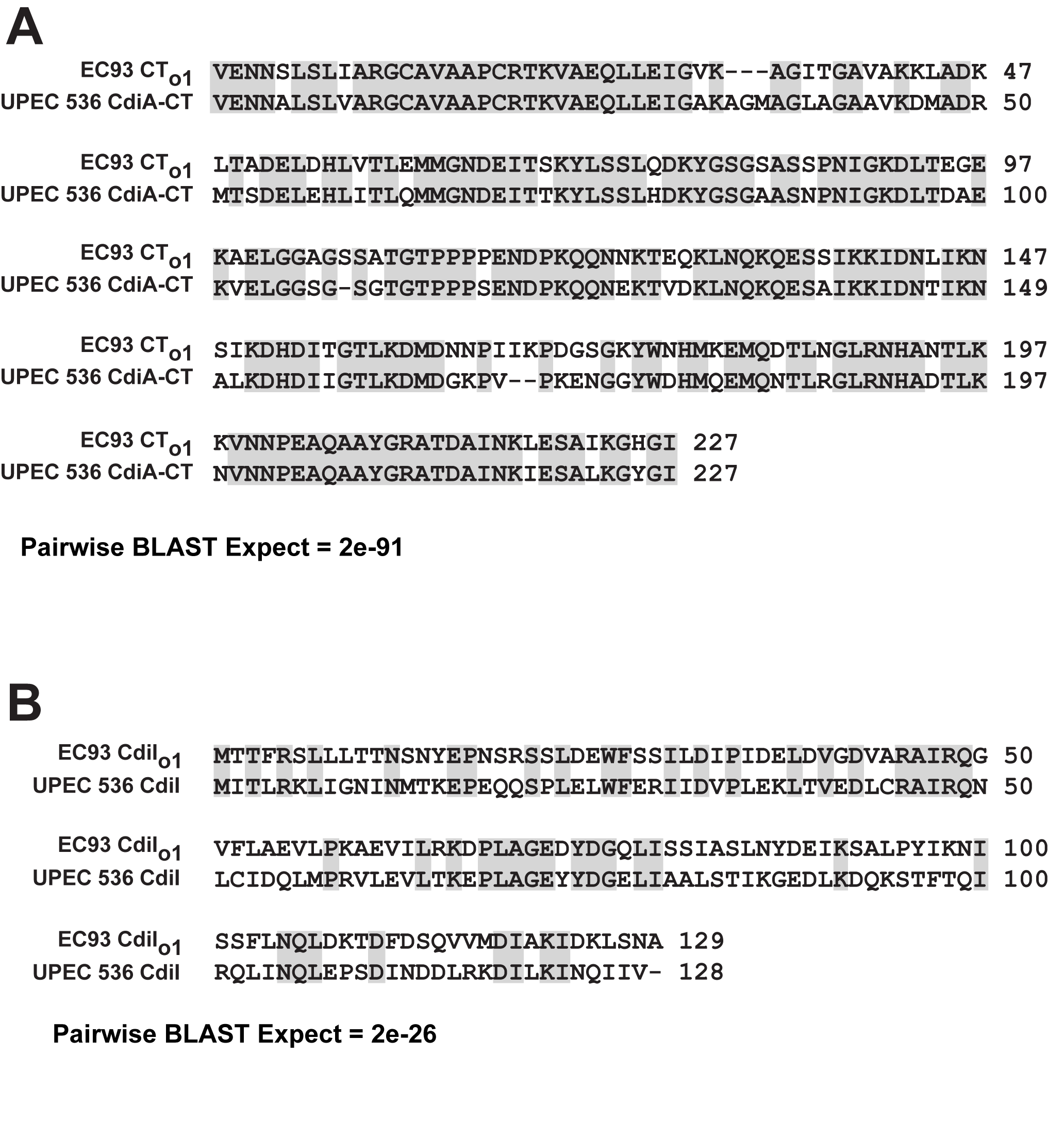

Supplement: Figure S1 — The EC93 orphan CdiA-CT/CdiI protein pair is related to the CdiA-CT/CdiI proteins from E. coli UPEC 536. A) Pairwise alignment of CdiA-CTo1 EC93 and CdiA-CTUPEC536 shows 76% sequence identity. Numbering begins at the Val residue of the conserved VENN motif. B) Alignment of CdiIo1 EC93 and CdiIUPEC536 shows 35% sequence identity. (TIF) [file pgen.1002217.s001.tif]

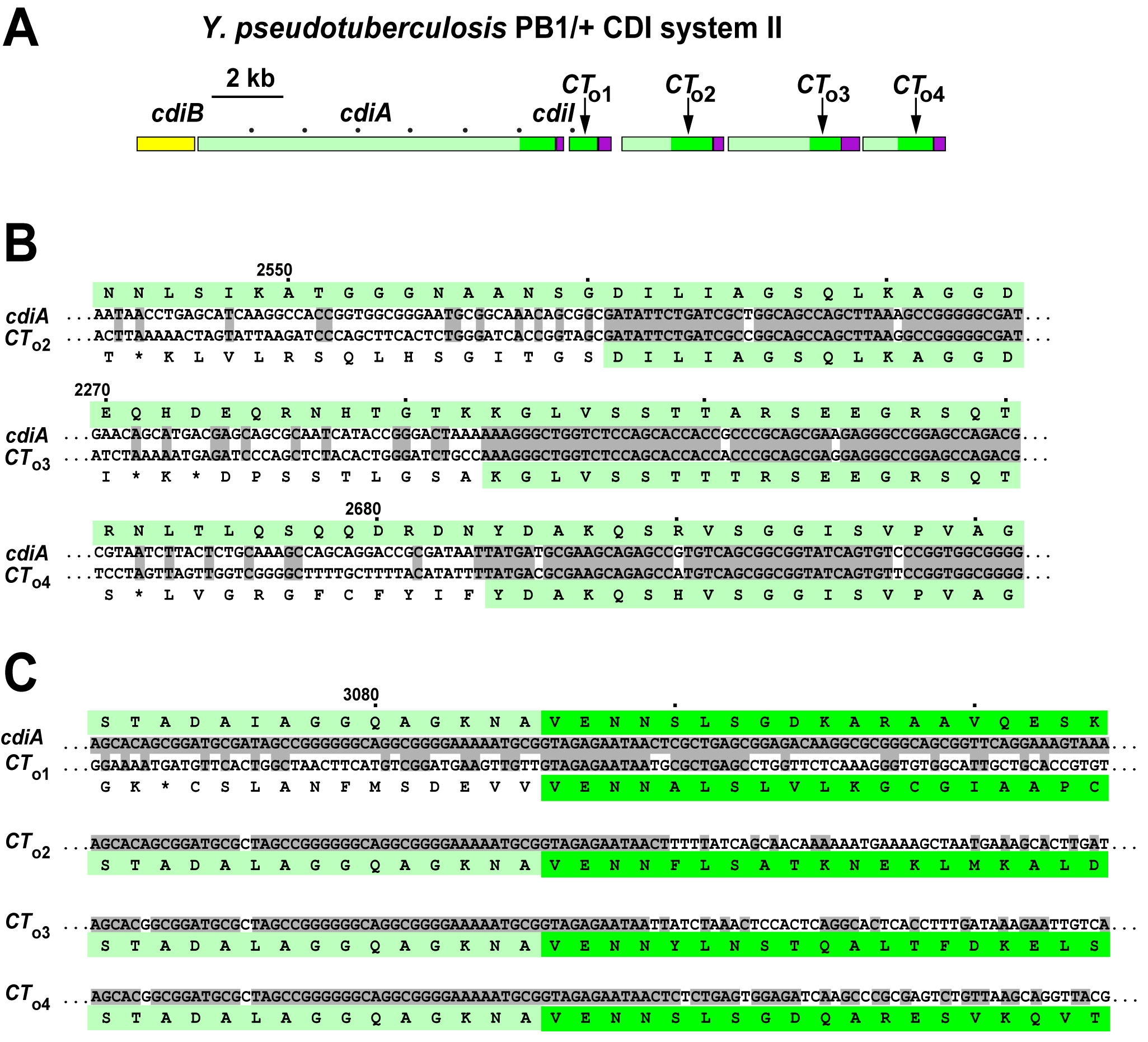

Supplement: Figure S2 — Orphan cdiA-CT fragments contain varying amounts of cdiA sequence upstream of the region encoding VENN. A) The region II cdi locus from Y. pseudotuberculosis PB1/+. CT o1 to CT o4 designate the orphan cdiA-CT o PB1(II) genes numbered according to their position in the locus. Dots above cdiA PB1(II) represent every 500 amino acids of the encoded protein. B) Pairwise alignments of the full-length Y. pseudotuberculosis PB1/+ region II cdiA gene with the linked orphan cdiA-CT sequences. The region where similarity with the full-length cdiA PB1(II) gene begins is shown for each orphan cdiA-CT sequence. Gray shading indicates nucleotide identity, and the numbers correspond to amino acid residues of the full-length CdiAPB1(II) protein. Amino acid sequences are given in one-letter code and asterisks (*) indicate termination codons. C) The nucleotide and predicted amino acid sequences for the VENN-encoding regions are presented. Orphan sequences shaded in gray are identical to that of the full-length cdiA PB1(II) gene. (TIF) [file pgen.1002217.s002.tif]

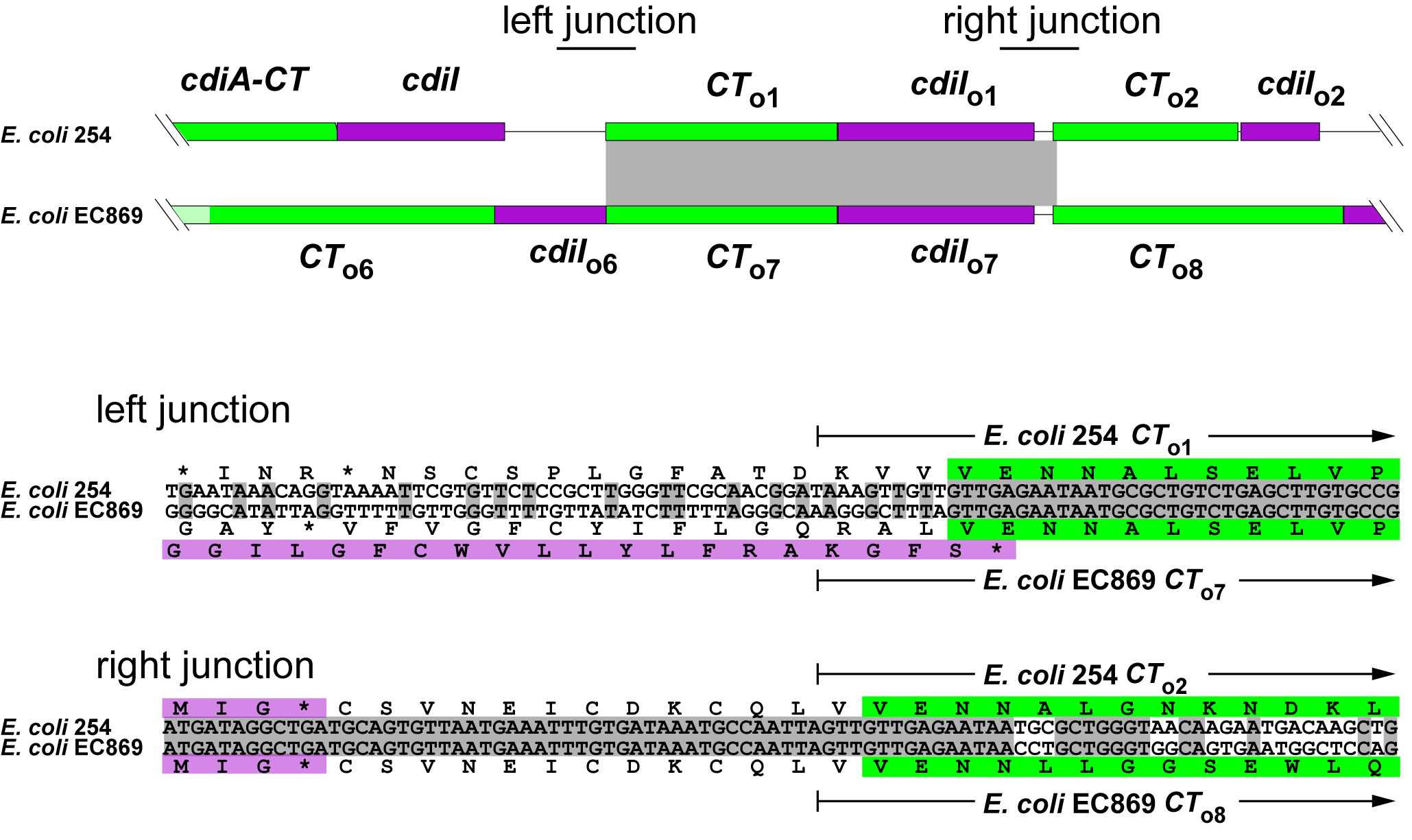

Supplement: Figure S4 — VENN-encoding sequences demarcate orphan cdiA-CT fragments. The orphan cdiA-CT o1 of E. coli 254 is related to orphan cdiA-CT o7 of E. coli EC869. The conserved region is shaded gray, and the left and right junctions of the conserved region are presented in detail. Conservation begins at the VENN-encoding regions for cdiA-CT o1 254 and cdiA-CT o7 EC869 (left junction) and extends through the predicted orphan cdiI genes to the VENN encoding regions of the following orphan cdiA-CT genes (99% sequence identity in 1048 nucleotides). The sequences then diverge immediately after VENN encoding regions cdiA-CT o2 254 and cdiA-CT o8 EC869. (TIF) [file pgen.1002217.s004.tif]

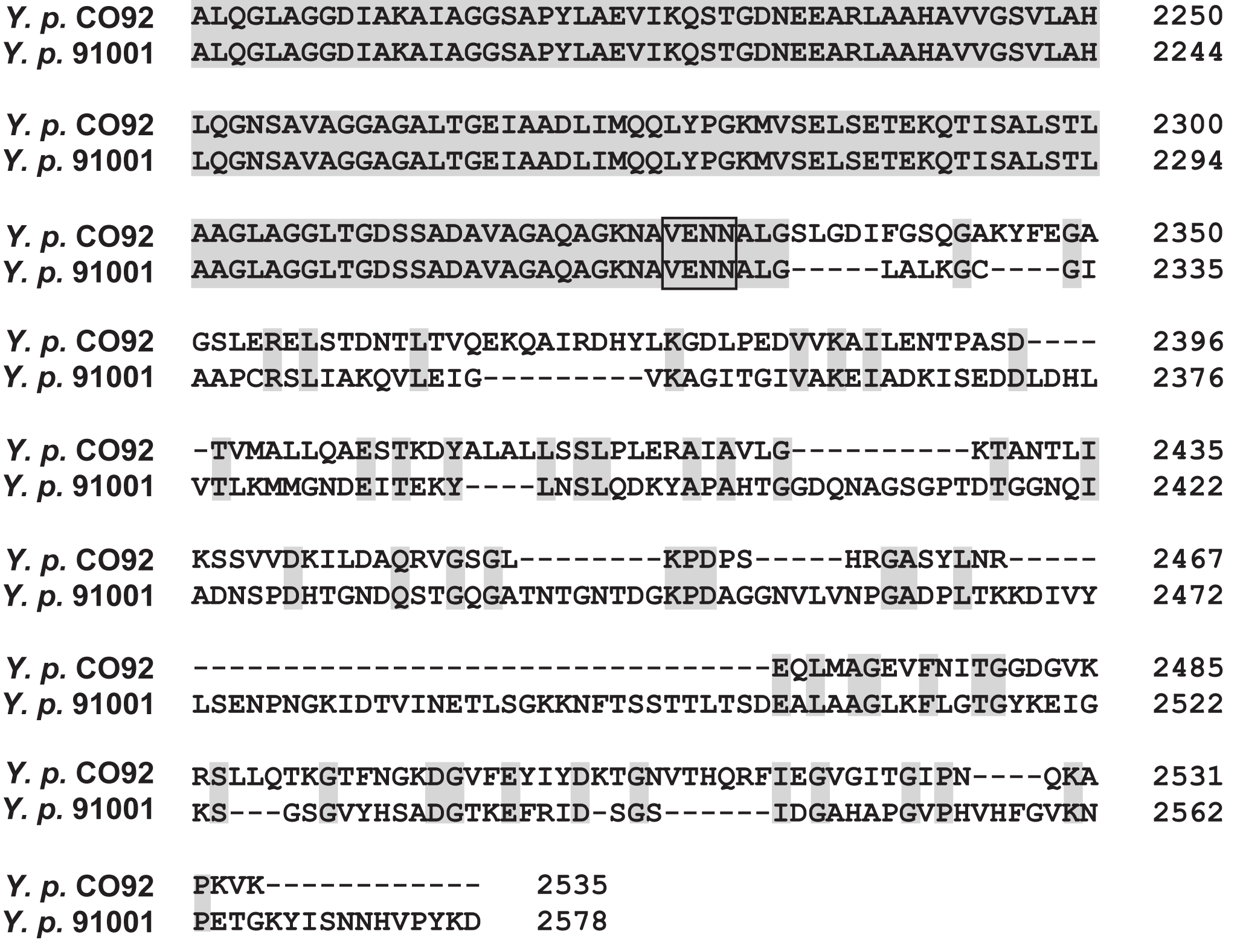

Supplement: Figure S5 — The CdiAI-CT of Y. pestis Microtus 91001 is unrelated to CdiA-CTs from other Y. pestis strains. Pairwise alignment of CdiA(I) proteins encoded by the region I cdi loci of Y. pestis Microtus 91001 and Y. pestis CO92. Numbers correspond to amino acid residues of the full-length predicted CdiA(I) proteins. Regions of sequence identity are shaded gray and the VENN peptide motif is boxed. (TIF) [file pgen.1002217.s005.tif]

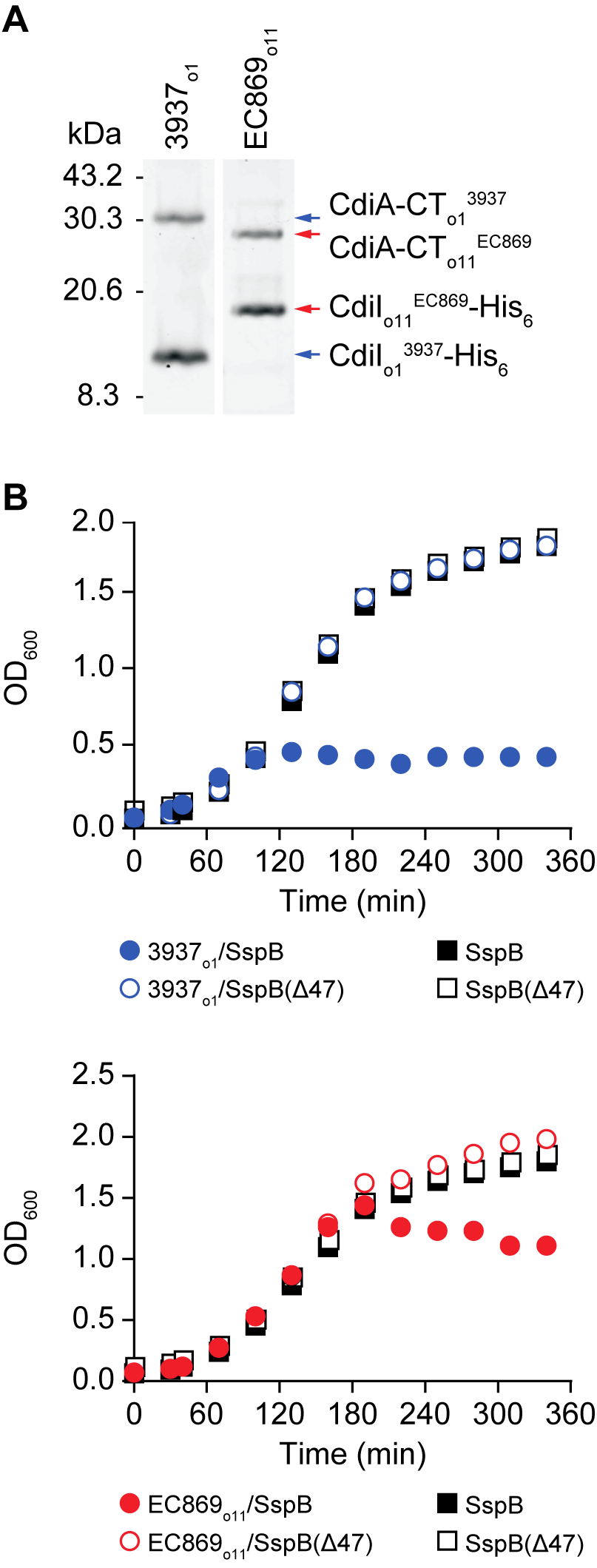

Supplement: Figure S6 — Orphan cdiA-CT/cdiI modules from D. dadantii 3937 and E. coli EC869 encode functional toxin/immunity pairs. A) Purification of orphan CdiA-CT/CdiI-His6 proteins. CdiA-CTo1 3937/CdiIo1 3937-His6 and CdiA-CTo11 EC869/CdiIo11 EC869-His6 complexes were purified by Ni2+-affinity chromatography under non-denaturing conditions and analyzed by SDS-PAGE. B) Growth curves of E. coli ΔsspB cells expressing orphan CdiA-CT/CdiI-DAS complexes. Degradation of CdiI-DAS proteins was initiated by the addition of L-arabinose to induce SspB synthesis. Control cells express SspB(Δ47), which does not deliver CdiI-DAS proteins to the ClpXP protease. Growth curves with square symbols represent control strains expressing SspB or SspB(Δ47), but not orphan CdiA-CT/CdiI-DAS complexes. (TIF) [file pgen.1002217.s006.tif]

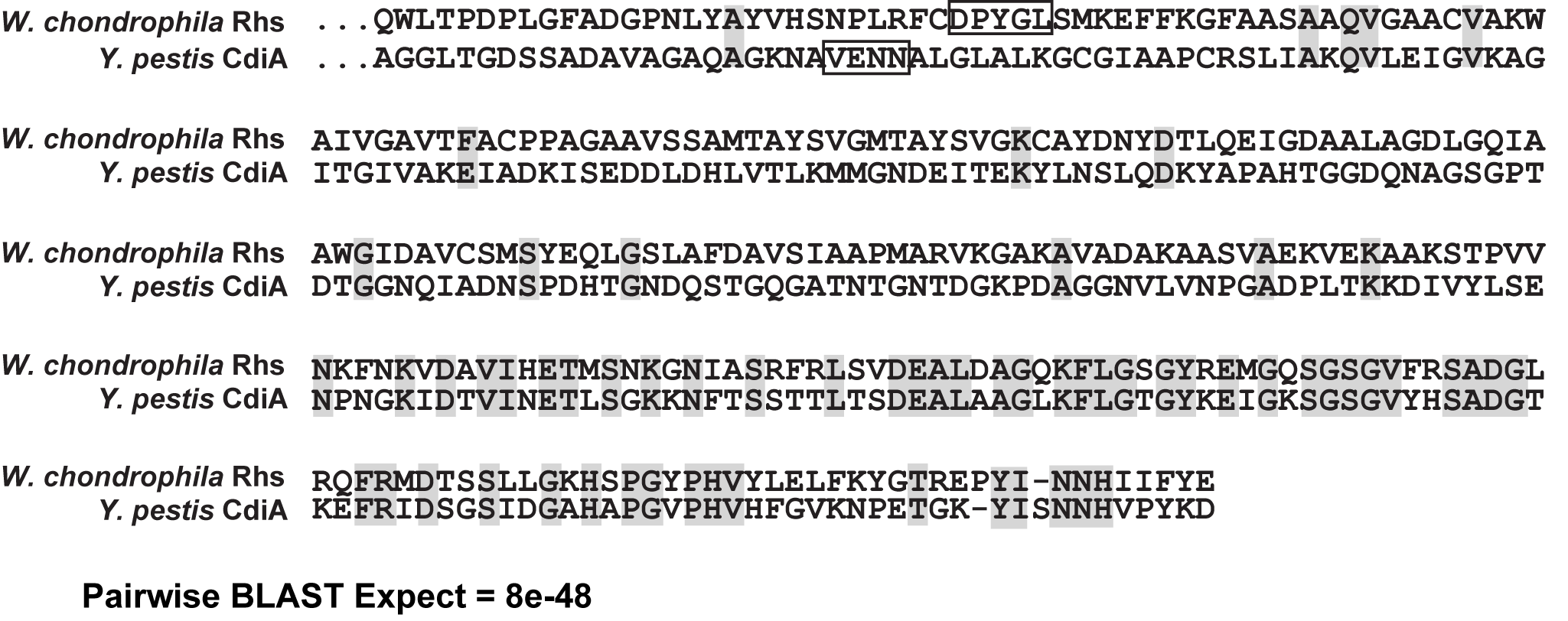

Supplement: Figure S7 — The CdiAI-CT of Y. pestis Microtus 91001 is related to an Rhs-CT from Waddlia chondrophila. Pairwise alignment of Y. pestis Microtus CdiA-CT91001(I) (Q74T84) and the C-terminal region of a predicted Rhs/YD-repeat protein from Waddlia chondrophila WSU 86–1044 (D6YTT8). Regions of sequence identity are shaded gray and the PxxxxDPxGL and VENN peptide motifs are boxed. (TIF) [file pgen.1002217.s007.tif]

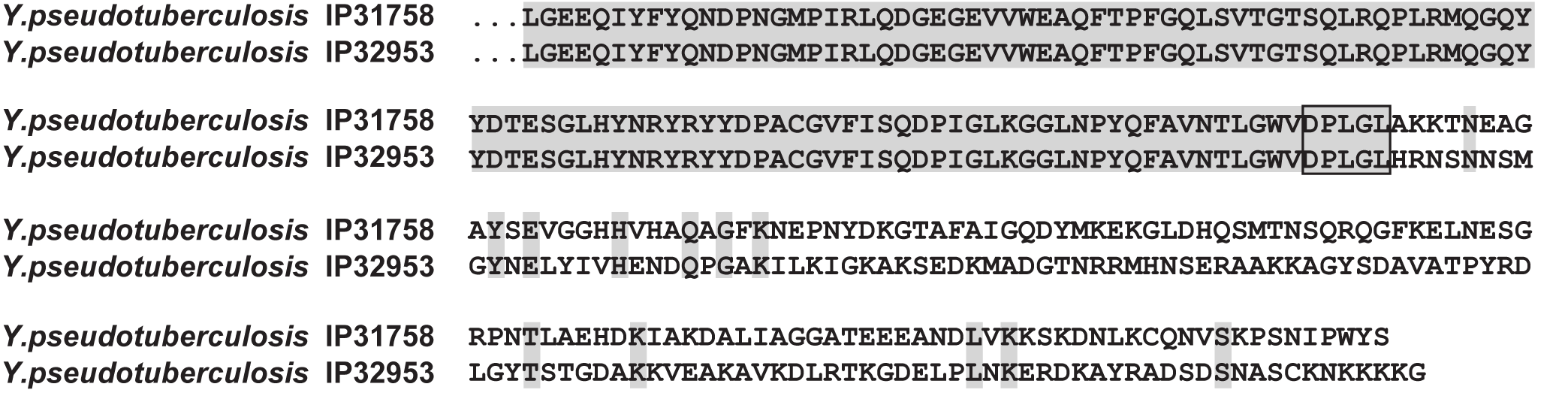

Supplement: Figure S8 — The C-terminal regions of Rhs proteins are variable. Pairwise alignment of related Rhs proteins from Y. pseudotuberculosis IP31758 and Y. pseudotuberculosis IP32953. Regions of identity are shaded gray. Sequences diverge abruptly after the DPxGL motif (boxed). (TIF) [file pgen.1002217.s008.tif]
