# Supplementary material for: Identification of Functional Toxin/Immunity Genes Linked to Contact-Dependent Growth Inhibition (CDI) and Rearrangement Hotspot (Rhs) Systems
Source: PLoS Genet. 2011 Aug 4;7(8):e1002217. doi: 10.1371/journal.pgen.1002217 (PMC3150448; doi:10.1371/journal.pgen.1002217)
Supplement: Figure S3 — Multiple sequence alignment of Y. pseudotuberculosis PB1/+ cdiA-CT nucleotide sequences. The nucleotide sequences encoding orphan cdiA-CTs from the Y. pseudotuberculosis PB1/+ region II cdi locus were aligned to the full-length cdiA PB1(II) gene. Numbering corresponds to the full-length CdiAPB1(II) protein sequence. Black indicates positions of sequence identity with the full cdiA PB1(II) gene. Sequence identity falls off abruptly after the VENN-encoding region (grey). (PDF) [file pgen.1002217.s003.pdf]

```
2272  ...D  E  Q  R  N  H  T  G  T  K  K  G  L  V  S  S  T  T  A
cdiA  CATGACGAGCAGCGCAATCATACCGGGACTAAAAAAGGGCTGGTCTCCAGCACCACCGCC

CTo3  AAATGAGATCCCAGCTCTACACTGGGATCTGCCAAAGGGCTGGTCTCCAGCACCACCACC


2292   R  S  E  E  G  R  S  Q  T  L  A  V  G  S  M  L  S  A  G  S
cdiA  CGCAGCGAAGAGGGCCGGAGCCAGACGCTGGCGGTGGGCTCGATGCTCTCGGCGGGTTCC

CTo3  CGCAGCGAGGAGGGCCGGAGCCAGACGCTGGCGGTGGGCTCGATGCTCTCGGCGGGTTCC


2312   I  D  V  S  S  Q  N  I  A  V  A  G  S  S  V  V  A  D  K  D
cdiA  ATTGATGTCAGTAGCCAAAATATCGCGGTCGCCGGCAGCAGCGTGGTGGCCGATAAGGAT

CTo3  ATTGATGTCAGTAGCCAAAATATCGCGGTCGCGGGCAGCAGCGTGGTGGCCGATAAGGAT


2332   I  R  L  R  A  Q  E  N  L  T  V  S  T  A  Q  Q  S  E  S  G
cdiA  ATTCGCCTGCGTGCGCAGGAAAACCTGACCGTGAGCACCGCGCAGCAGAGCGAGAGCGGG

CTo3  ATTCGCCTGCGTGCGCAGGAAAACCTGACCGTGAGCACCGCGCAGCAGAGCGAGAGCGGG


2352   S  Q  L  F  E  Q  K  K  S  G  L  M  S  T  G  G  I  G  V  F
cdiA  TCTCAGCTATTCGAGCAGAAAAAATCCGGCCTGATGAGCACCGGCGGTATCGGTGTCTTT

CTo3  TCTCAGCTATTCGAGCAGAAAAAATCCGGCCTGATGAGCACCGGCGGTATCGGTGTCTTT


2372   I  G  T  S  R  Q  K  T  T  D  Q  T  Q  T  V  S  H  I  G  S
cdiA  ATCGGGACTTCCCGGCAGAAAACCACCGACCAGACCCAAACGGTGAGCCATATTGGCAGC

CTo3  ATCGGGACTTCCCGGCAGAAAACCACCGACCAGACCCAAACGGTGAGCCATATTGGCAGC


2392   T  V  G  S  L  T  G  N  V  R  L  E  A  G  N  Q  L  A  L  H
cdiA  ACGGTGGGCAGCCTGACGGGCAATGTGCGTCTCGAGGCGGGCAATCAGTTAGCCCTTCAC

CTo3  ACGGTGGGCAGCCTGACGGGCAATGTGCGTCTCGAGGCGGGCGATCAGTTAGCCCTTCAC


2412   G  S  D  V  V  A  G  K  D  L  A  L  T  G  A  D  V  A  I  S
cdiA  GGCAGCGACGTGGTGGCGGGTAAAGACCTCGCCCTGACAGGGGCGGATGTCGCGATCAGC

CTo3  GGCAGCGACGTGGTGGCGGGTAAAGACCTCGCCCTGACAGGGGCGGATGTCGCGATCAGC


2432   A  A  E  N  S  R  S  Q  Q  Y  T  A  E  S  K  Q  S  G  L  T
cdiA  GCCGCAGAAAATAGCCGTTCTCAACAGTATACTGCCGAGAGTAAACAAAGTGGCCTGACG

CTo3  GCCGCAGAAAATAGCCGTTCTCAACAGTATACTGCCGAGAGTAAACAAAGTGGCCTGACG


2452   V  A  L  S  G  P  V  G  S  A  V  N  T  A  V  T  T  A  K  A
cdiA  GTGGCGCTGTCCGGACCGGTCGGCAGTGCCGTCAATACGGCGGTCACCACCGCCAAAGCG

CTo3  GTGGCGCTGTCCGGACCGGTCGGCAGTGCCGTCAATACGGCGGTCACCACCGCCAAAGCG


2472   A  R  E  E  N  T  G  R  L  A  G  L  Q  G  V  K  A  A  L  S
cdiA  GCCCGCGAAGAAAACACCGGCCGGCTGGCGGGATTGCAAGGGGTCAAAGCGGCGCTGTCG

CTo3  GCCCGCGAAGAAAACACCGGGCGGCTGGCGGGATTGCAAGGGGTCAAAGCGGCGCTGTCG
```

```
2492  G  V  Q  A  V  Q  A  G  Q  L  V  Q  A  Q  G  G  G  V  T  E
cdiA  GGGGTGCAAGCGGTGCAGGCCGGGCAACTGGTGCAGGCCCAGGGAGGCGGAGTCACTGAG

CTo3  GGGGTGCAGGCGGTGCAGGCCGGGCAACTGGTGCAGGCGCAGGGAGGTGGTGTTGCTGAG


2512  M  V  G  V  S  V  S  L  G  S  Q  K  S  S  S  Q  Q  Q  Q  E
cdiA  ATGGTGGGCGTCAGTGTGTCGTTAGGCTCACAGAAATCGTCCTCGCAGCAACAGCAGGAA

CTo3  ATGGTGGGCGTCAGTGTGTCGTTAGGCTCACAAAAATCGTCCTCGCAGCAACAGCAGGAA


2532  Q  T  Q  V  S  G  S  A  L  T  A  G  N  N  L  S  I  K  A  T
cdiA  CAGACCCAGGTGAGCGGTTCGGCCCTGACGGCGGGTAATAACCTGAGCATCAAGGCCACC
CTo2                                  ...ACTTAAAAACTAGTATTAAGATCC
CTo3  CAGACCCAGGTGAGCGGTTCGGCCCTGACGGCGGGTAATAACCTGAGCATCAAGGCCACC


2552  G  G  G  N  A  A  N  S  G  D  I  L  I  A  G  S  Q  L  K  A
cdiA  GGTGGCGGGAATGCGGCAAACAGCGGCGATATTCTGATCGCTGGCAGCCAGCTTAAAGCC
CTo2  CAGCTTCACTCTGGGATCACCGGTAGCGATATTCTGATCGCCGGCAGCCAGCTTAAGGCC
CTo3  GGTGGCGGGAATGCGGCAAACAGCGGCGATATTCTGATCGCTGGCAGCCAGCTTAAAGCC


2572  G  G  D  T  R  L  D  A  A  R  D  V  R  L  L  G  A  A  N  R
cdiA  GGGGGCGATACCCGGCTTGATGCGGCGCGTGACGTGCGGTTACTCGGCGCGGCCAATAGG
CTo2  GGGGGCGATACCCGGCTTGATGCGGCGCGTGACGTGCAGTTACTCGGCGCGGCCAATAGG
CTo3  GGGGGCGATACCCGGCTTGATGCGGCGCGTGACGTGCGGTTACTCGGCGCGGCCAATAGG


2592  Q  K  T  D  G  S  N  S  S  R  G  G  S  V  G  V  S  V  G  G
cdiA  CAAAAAAACCGACGGCAGTAACAGCAGCCGTGGCGGTAGCGTTGGCGTCAGTGTGGGGGGC
CTo2  CAAAAAAACCGACGGCAGTAACAGCAGCCGTGGCGGTAGCGTTGGCGTCAGTGTGGGGGGC
CTo3  CAAAAAAACCGACGGCAGTAACAGCAGCCGTGGCGGTAGCGTTGGCGTCAGTGTGGGGGGC


2612  S  G  L  S  V  F  A  N  A  N  K  G  Q  G  N  E  R  G  D  G
cdiA  AGCGGTCTGAGCGTCTTTGCCAATGCCAACAAGGGGCAGGGCAATGAGCGCGGTGACGGC
CTo2  AGCGGTCTGAGCGTCTTTGCCAATGCCAACAAGGGGCAGGGCAATGAGCGCGGTGACGGC
CTo3  AGCGGTCTGAGCGTCTTTGCCAATGCCAACAAGGGGCAGGGCAATGAGCGCGGTGACGGC


2632  T  F  W  T  E  T  T  V  D  S  G  G  M  F  S  L  R  S  G  R
cdiA  ACTTTCTGGACGGAAACCACCGTCGACAGTGGCGGAATGTTCTCGTTGCGCAGTGGCCGC
CTo2  ACTTTCTGGACGGAAACCACCGTCGACAGTGGCGGAATGTTCTCGTTGCGCAGCGGTCGC
CTo3  ACTTTCTGGACGGAAACCACCGTCGACAGTGGCGGAATGTTCTCGTTGCGCAGCGGCCGC


2652  D  T  A  L  T  G  A  Q  V  S  A  E  T  V  K  A  D  V  G  R
cdiA  GATACGGCACTGACCGGCGCGCAGGTCAGCGCTGAAACGGTCAAGGCCGATGTGGGGCGT
CTo2  GATACGGCACTGACCGGCGCGCAGGTCAGCGCTGAAACGGTCAAGGCCGATGTGGGGCGT
CTo3  GATACGGCACTGACCGGCGCGCAGGTCAGCGCTGAAACGGTCAAGGCCGATGTGGGGCGT
CTo4                                                       ...TCC

2672  N  L  T  L  Q  S  Q  Q  D  R  D  N  Y  D  A  K  Q  S  R  V
cdiA  AATCTTACTCTGCAAAGCCAGCAGGACCGCGATAATTATGATGCGAAGCAGAGCCGTGTC
CTo2  AATCTTACTCTGCAAAGCCAGCAGGACCGCGATAATTATGATGCGAAGCAGAGCCATGTC
CTo3  AATCTTACTCTGCAAAGCCAGCAGGACCGCGATAATTATGATGCGAAGCAGAGCCGTGCC
CTo4  TAGTTAGTTGGTCGGGGCTTTTGCTTTTACATATTTTATGACGCGAAGCAGAGCCATGTC

2692  S  G  G  I  S  V  P  V  A  G  G  G  A  A  V  N  L  S  M  S
cdiA  AGCGGCGGTATCAGTGTCCCGGTGGCGGGGGGCGGTGCCCGCGGTCAACCTGAGCATGAGC
CTo2  AGCGGCGGTATCAGTGTCCCGGTGGCGGGGGGCGGTGCCCGCGGTCAACCTGAGCATGAGC
CTo3  AGCGGCGGTATCAGTGTCCCAGTAGCGGGGGGCGGTGCCCGCGGTCAACCTGAGCATGAGC
CTo4  AGCGGCGGTATCAGTGTTCCGGTGGCGGGGGGCGGTGCCCGCGGTCAACCTGAGCATGAGC
```

```
2712   D  R  L  S  S  Q  Y  D  S  V  Q  A  Q  T  G  I  F  A  G
cdiA  CGTGACAGGTTATCCAGCCAGTATGATTCGGTGCAGGCGCAGACGGGTATTTTTGCCGGC
CTo2  CGTGACAGGTTATCCAGCCAGTATGATTCGGTGCAGGCGCAGACGGGTATTTTTGCCGGC
CTo3  CGTGACAGGCTATCCAGCCAGTATGACTCGGTGCAGGCGCAGACGGGTATTTTTGCCGGT
CTo4  CGTGACAGGTTATCCAGCCAGTATGATTCGGTGCAGGCGCAGACGGGTATTTTTGCCGGC

2732  S  G  G  V  D  I  R  V  G  E  H  T  Q  L  D  G  A  V  I  A
cdiA  TCTGGCGGTGTTGATATCCGGGTGGGGGAGCACACCCAACTGGATGGCGCGGTGATTGCC
CTo2  TCTGGCGGTGTTGATATCCGGGTGGGTGAGCACACCCAGTTGGATGGCGCGGTGATTGCC
CTo3  TCTGGCGGTGTTGATATCCGGGTGGGGGAGCACACCCAACTGGATGGCGCGGTGATTGCC
CTo4  TCTGGCGGTGTTGATATCCGGGTGGGGGAGCACACCCAACTGGATGGCGCGGTGATTGCC

2752  S  T  A  A  A  D  K  N  T  L  D  T  G  T  L  G  F  S  D  I
cdiA  AGCACGGCGGCAGCCGATAAAAACACGCTGGATACCGGCACACTGGGCTTCAGTGATATC
CTo2  AGCACGGCGGCAGCCGATAAAAACACGCTGGATACCGGCACACTGGGCTTCAGTGATATC
CTo3  AGCACGGCGGCAGCCGATAAAAACACGCTGGATACCGGCACACTGGGCTTCAGTGATATC
CTo4  AGCACGGCGGCAGCCGATAAAAACACGCTGGATACCGGCACACTGGGCTTCAGTGATATC

2772  K  N  K  A  V  F  T  V  E  H  Q  G  G  S  L  S  T  G  G  P
cdiA  AAAAATAAAGCGGTATTCACGGTAGAGCATCAGGGCGGCAGCCTGAGCACCGGTGGCCCG
CTo2  AAAAATAAAGCGGTATTCACGGTAGAGCATCAGGGCGGCAGCCTGAGCACCGGTGGCCCG
CTo3  AAAAATAAAGCGGTATTCACGGTGGAGCATCAGGGCGGCAGCCTGAGCACCGGTGGCCCG
CTo4  AAAAATAAAGCGGTATTCACGGTGGAGCATCAGGGCGGCAGCCTGAGCACCGGTGGCCCG

2792  V  G  S  D  L  L  S  N  L  G  G  M  V  L  A  G  L  G  N  G
cdiA  GTGGGGTCAGACCTGCTGAGTAATCTGGGCGGTATGGTGCTCGCGGGGCTGGGCAATGGC
CTo2  GTGGGGTCAGACCTGCTGAGTAATCTGGGCGGCATGGTGCTCGCGGGGCTGGGCAATGGC
CTo3  GTGGGGTCAGACCTGCTGAGTAATCTGGGCGGCATGGTGCTCGCGGGGCTGGGCAATGGC
CTo4  GTGGGGTCAGACCTGCTGAGTAATCTGGGTGGCATGGTGCTCGCGGGGCTGGGCAATGGC

2812  G  Y  A  E  G  T  T  Q  A  A  V  S  E  G  T  I  T  V  R  D
cdiA  GGATATGCCGAAGGCACCACGCAGGCGGCAGTGAGTGAGGGCACGATTACCGTTCGCGAC
CTo2  GGATATGCTGAAGGCACCACGCAGGCGGCAGTGAGTGAGGGCACGATTACCGTTCGCGAC
CTo3  GGATATGCCGAAGGCACCACGCAGGCGGCAGTGAGTGAGGGCACGATTACCGTTCGCGAC
CTo4  GGATATGCCGAAGGCACCACGCAGGCGGCAGTGAGTGAGGGCACGATTACCGTTCGCGAC

2832  T  E  N  Q  Q  Q  N  V  D  D  L  S  R  D  T  G  N  A  N  G
cdiA  ACGGAGAATCAACAGCAGAATGTTGATGACCTGAGCCGGGACACCGGGAATGCCAACGGC
CTo2  ACGGAGAATCAACAGCAGAATGTTGATGACCTGAGCCGGGACACCGGGAATGCCAACGGC
CTo3  ACGGAGAATCAACAGCAGAATGTTGATGACCTGAGCCGGGACACCGGGAATGCCAACGGC
CTo4  ACGGAGAATCAACAGCAGAATGTTGATGACCTGAGCCGGGACACCGGGAATGCCAACGGC

2852  S  I  G  P  I  F  D  K  E  K  E  Q  N  R  L  K  E  V  Q  L
cdiA  AGTATCGGGCCGATTTTTGATAAAGAGAAAGAGCAGAACCGGCTGAAAGAAGTGCAGCTG
CTo2  AGTATCGGGCCGATTTTTGATAAAGAGAAAGAGCAGAACCGACTGAAAGAAGTGCAGCTG
CTo3  AGTATCGGGCCGATTTTTGATAAAGAGAAAGAGCAGAACCGGCTGAAAGAAGTGCAGCTG
CTo4  AGTATCGGGCCGATTTTTGATAAAGAGAAAGAGCAGAACCGGCTGAAAGAAGTGCAGCTG

2872  I  G  E  I  G  G  Q  A  L  D  I  A  S  T  Q  G  K  I  I  A
cdiA  ATTGGCGAGATAGGCGGTCAGGCGTTAGATATTGCCTCCACGCAGGGCAAGATAATTGCC
CTo2  ATTGGCGAGATAGGCGGTCAGGCGTTAGATATTGCCTCCACGCAGGGCAAGATAATTGCC
CTo3  ATTGGCGAGATAGGCGGTCAGGCGTTAGATATTGCCTCCACGCAGGGCAAGATAATTGCC
CTo4  ATTGGCGAGATAGGCGGTCAGGCGCTGGATATTGCCTCCACGCAGGGCAAGATAATTGCC

2892  T  H  A  A  N  D  K  M  K  A  V  K  P  E  D  I  A  T  A  E
cdiA  ACTCACGCGGCAAACGACAAGATGAAGGCGGTGAAGCCGGAAGATATCGCCACGGCGGAA
CTo2  ACTCACGCGGCAAACGACAAGATGAAGGCGGTGAAGCCGGAAGATATCGCCGCGGCGGAA
CTo3  ACTCACGCGGCAAACGACAAGATGAAGGCGGTGAAGCCGGAAGATATCGCCGCGGCGGAA
CTo4  ACTCACGCGGCAAACGACAAGATGAAGGCGGTGAAGCCGGAAGATATCGTCGCGGCGGAA

2912  K  Q  W  E  K  A  H  P  G  K  A  A  T  A  E  D  I  N  Q  Q
cdiA  AAACAGTGGGAGAAAGCCCATCCGGGCAAGGCGGCCACGGCAGAGGACATCAACCAGCAG
CTo2  AAACAGTGGGAGAAAGCCCATCCGGGCAAGGCGGCCACGGCAGAGGACATCAACCAGCAG
CTo3  AAACAGTGGGAGAAAGCCCATCCGGGCAAGGCGGCCACGGCAGAGGACATCAACCAGCAG
CTo4  AAACAGTGGGAGAAAGCCAATCCGGGCAAGGCGGCCACGGCAGAGGACATCAACCAGCAG
```

```
2932  I  Y  Q  T  A  Y  N  Q  A  F  N  D  S  G  F  G  T  G  G  P
cdiA  ATTTACCAGACGGCATATAATCAGGCATTTAACGACTCAGGATTTGGGACCGGCGGCCCG
CTo2  ATTTACCAGACGGCATATAATCAGGCATTTAACGAGTCAGGATTTGGGACAGGCGGCCCG
CTo3  ATTTACCAGACGGCGTATAATCAGGCATTTAACGACTCAGGATTTGGGACCGGCGGCCCG
CTo4  ATTTACCAGACGGCGTATAATCAGGCGTTTAACGCGTCAGGATTTGGGACCGGCGGCCCG

2952  V  Q  R  G  M  Q  A  A  T  A  A  V  Q  G  L  A  G  G  N  L
cdiA  GTGCAACGCGGGATGCAGGCGGCGACAGCCGCCGTGCAGGGGCTGGCTGGCGGGAATCTG
CTo2  GTGCAACGCGGTATGCAGGCGGCGACAGCCGCCGTGCAGGGGCTGGCCGGCGGGAATATG
CTo3  GTGCAACGCGGGATGCAGGCGGCGACAGCCGCCGTGCAGGGGCTGGCCGGCGGGAATCTG
CTo4  GTGCAACGCGGGATGCAGGCGGCGACAGCCGCCGTGCAGGGGCTGGCCGGCGGGAATATG

2972  G  A  A  L  T  G  A  S  A  P  Y  L  A  G  V  I  K  Q  S  T
cdiA  GGTGCGGCCCTGACGGGTGCCAGTGCGCCGTATCTAGCGGGGGTGATTAAGCAAAGTACA
CTo2  GGTGCGGCCCTGACGGGTGCCAGTGCGCCGTATCTGGCGGGGGTGATTAAGCAAAGTACA
CTo3  GGTGCGGCCCTGACGGGTGCCAGTGCGCCGTATCTGGCGGGGGTGATTAAGCAAAGTACG
CTo4  GGTGCGGCCCTGACGGGTGCCAGTGCGCCGTATCTGGCGGGGGTGATTAAGCAAAGTACG

2992  G  D  N  P  A  A  N  T  M  A  H  A  V  L  G  A  V  T  A  Y
cdiA  GGCGATAATCCGGCGGCTAACACAATGGCACACGCGGTATTGGGCGCGGTGACGGCCTAT
CTo2  GGCGATAATCCGGCGGCCAACACAATGGCACACGCGGTATTGGGCGCGGTGACTGCCTAT
CTo3  GGCGATAATCCGGCGGCCAACACAATGGCACACGCGGTATTGGGCGCGGTGACGGCCTAT
CTo4  GGCGATAATCCGGCGGCCAACACAATGGCACACGCGGTATTGGGCGCGGTGACGGCCTAT

3012  A  R  G  N  N  A  L  A  G  A  A  G  A  A  T  A  E  L  M  A
cdiA  GCCCGTGGCAACAATGCGCTGGCGGGTGCGGCGGGCGCGGCCACGGCGGAGTTGATGGCC
CTo2  GCCCGTGGCAACCATGCGCTGGCGGGTGCGGCTGGCGCGGCCACGGCGGAGTTGATGGCC
CTo3  GCCAGCGGCAACCATGCGCTGGCGGGTGCGGCGGGCGCGGCCACGGCGGAGTTGATGGCC
CTo4  GCCAGCGGCAACCATGCGCTGGCGGGTGCGGCGGGCGCGGCCACGGCGGAGTTGATGGCC

3032  P  T  I  I  S  A  L  G  W  D  K  N  T  L  T  E  G  Q  K  Q
cdiA  CCCACGATTATCAGCGCGCTGGGCTGGGACAAGAACACACTCACCGAAGGTCAGAAACAG
CTo2  CCCACGATTATCAGCGCGCTGGGCTGGGACAAGAACACACTCACTGAAGGTCAGAAACAG
CTo3  CCCACGATTATCAGCGCGCTGGGCTGGGACAAGAACACACTCACCGAAGGTCAGAAACAG
CTo4  CCCACGATTATCAGCGCGCTGGGCTGGGACAAGAACACACTCACCGAAGGTCAGAAACAG

3052  A  V  S  A  L  S  T  L  A  A  G  L  A  G  G  L  T  G  D  S
cdiA  GCTGTCAGCGCACTGAGTACATTAGCCGCCGGGCTGGCCGGTGGTTTGACAGGTGACAGC
CTo2  GCTGTCAGCGCACTGAGTACATTAGCCGCCGGGCTGGCCGGTGGCCTGACATGTGACAGC
CTo3  GCTGTCAGCGCACTGAGTACATTAGCCGCCGGGCTGGCCGGTGGTTTGACAGGTGACAGC
CTo4  GCTGTCAGCGCACTGAGTACATTAGCCGCCGGGCTGGCCGGTGGTTTGACAGGTGACAGC

3072  T  A  D  A  I  A  G  G  Q  A  G  K  N  A  V  E  N  N  S  L
cdiA  ACAGCGGATGCGATAGCCGGGGGGCAGGCGGGGAAAAATGCGGTAGAGAATAACTCGCTG
CTo2  ACAGCGGATGCGCTAGCCGGGGGGCAGGCGGGGAAAAATGCGGTAGAGAATAACTTTTTA
CTo3  ACGGCGGATGCGCTAGCCGGGGGGCAGGCGGGGAAAAATGCGGTAGAGAATAATTATCTA
CTo4  ACGGCGGATGCGCTAGCCGGGGGGCAGGCGGGGAAAAATGCGGTAGAGAATAACTCTCTG

3092  S  G  D  K  A  R  A  A  V  Q  E  S  K...
cdiA  AGCGGAGACAAGGCGCGGGCAGCGGTTCAGGAAAGTAAA
CTo2  TCAGCAACAAAAAATGAAAAGCTAATGAAAGCACTTGAT
CTo3  AACTCCACTCAGGCACTCACCTTTGATAAAGAATTGTCA
CTo4  AGTGGAGATCAAGCCCGCGAGTCTGTTAAGCAGGTTACG
```
